# Characterization of Minimizers of Aviles–Giga Functionals in Special Domains

**DOI:** 10.1007/s00205-021-01704-w

**Published:** 2021-08-23

**Authors:** Elio Marconi

**Affiliations:** EPFL B, Station 8, 1015 Lausanne, Switzerland

## Abstract

We consider the singularly perturbed problem $F_\varepsilon (u,\Omega ):=\int _\Omega \varepsilon |\nabla ^2u|^2 + \varepsilon ^{-1}|1-|\nabla u|^2|^2$ on bounded domains $\Omega \subset {\mathbb {R}}^2$. Under appropriate boundary conditions, we prove that if $\Omega$ is an ellipse, then the minimizers of $F_\varepsilon (\cdot ,\Omega )$ converge to the viscosity solution of the eikonal equation $|\nabla u|=1$ as $\varepsilon \rightarrow 0$.

## Introduction

### The Main Result

We consider the family of functionals

1.1 $$\begin{aligned} F_\varepsilon (u,\Omega ):= \int _{\Omega } \left( \varepsilon |\nabla ^2 u|^2 + \frac{1}{\varepsilon }\left| 1-|\nabla u|^2\right| ^2\right) dx, \end{aligned}$$

where $\Omega \subset {\mathbb {R}}^2$ is a $C^2$ bounded open set, $\varepsilon >0$ and $u \in W^{2,2}_0(\Omega )$. These functionals were introduced in [4] and proposed as a model for blistering in [27]. In these cases we are interested in the minimizers $u_\varepsilon$ of $F_\varepsilon$ in the space

$$\begin{aligned} \Lambda (\Omega ):= \left\{ u\in W^{2,2}_0(\Omega ) : \frac{\partial u}{\partial n}=-1 \text{ on } \partial \Omega \right\} , \end{aligned}$$

where *n* denotes the outer normal to $\Omega$. The final goal is the understanding of the behavior of $u_\varepsilon$ as $\varepsilon \rightarrow 0$. In [27] (and more explicitly in [5]) it is conjectured that

1.2 $$\begin{aligned} u_\varepsilon \rightarrow {\bar{u}}:= {{\,\mathrm{dist}\,}}(\cdot , \partial \Omega ), \end{aligned}$$

at least for convex domains $\Omega$. A first partial result in this direction was obtained in [16, Theorem 5.1], where the authors proved that if $\Omega$ is an ellipse, then

1.3 $$\begin{aligned} \lim _{\varepsilon \rightarrow 0} \min F_{\varepsilon }(\cdot ,\Omega ) = F_0({\bar{u}}, \Omega ), \end{aligned}$$

where $F_0$ is the candidate asymptotic functional that we are going to introduce in (1.4).

The main result of this paper is the proof of (1.2) in the same setting as in [16], namely

#### Theorem 1.1

Let $\Omega \subset {\mathbb {R}}^2$ be an ellipse and, for every $\varepsilon >0$, let $u_\varepsilon$ be a minimizer of $F_\varepsilon (\cdot , \Omega )$. Then

$$\begin{aligned} \lim _{\varepsilon \rightarrow 0}u_{\varepsilon } = {{\,\mathrm{dist}\,}}(\cdot , \partial \Omega ) \qquad \text{ in } W^{1,1}(\Omega ). \end{aligned}$$

This result is obtained as a corollary after showing that ${\bar{u}}$ is the unique minimizer of a suitable asymptotic problem for $F_\varepsilon (\cdot , \Omega )$ as $\varepsilon \rightarrow 0$. In order to rigorously introduce it, we recall some previous results (see also the introduction of [10] for a presentation of the history of the problem).

### Previous Results

In what follows, $\Omega$ denotes a $C^2$ bounded open subset of ${\mathbb {R}}^2$. Independently from the validity of 1.2, it is conjectured already in [4] that

1. if $u_\varepsilon$ is such that $\limsup _{\varepsilon \rightarrow 0}F_\varepsilon (u_\varepsilon ,\Omega )< \infty$, then $u_\varepsilon$ converges up to subsequences to a Lipschitz solution *u* of the eikonal equation $|\nabla u|=1$;
2. if $u_\varepsilon$ is a sequence of minimizers of $F_\varepsilon (\cdot , \Omega )$, then any limit *u* of $u_\varepsilon$ minimizes the functional
  1.4 $$\begin{aligned} F_0(v,\Omega ):= \frac{1}{3}\int _{J_{\nabla v}}|\nabla v ^+ - \nabla v^-|^3 d{\mathcal {H}}^1, \end{aligned}$$
  among the solutions of the eikonal equation. Here, $J_{\nabla v}$ denotes the jump set of $\nabla v$ and $\nabla v^\pm$ the corresponding traces.

A positive answer to the first point was obtained independently in [12] and [2]. A fundamental notion in this analysis and in particular in [12] is the one of entropy, borrowed from the field of conservation laws.

#### Definition 1.2

We say that $\Phi \in C^\infty _c({\mathbb {R}}^2;{\mathbb {R}}^2)$ is an *entropy* if for every open set $\Omega \subset {\mathbb {R}}^2$ and every smooth $m:\Omega \rightarrow {\mathbb {R}}^2$ it holds that

1.5 $$\begin{aligned} \left( \mathrm {div}\,m=0 \text{ and } |m|^2=1 \right) \quad \Rightarrow \quad \mathrm {div}(\Phi (m))=0. \end{aligned}$$

We will denote by ${\mathcal {E}}$ the set of entropies.

We will consider the following family of entropies introduced first in [6, 16]:

$$\begin{aligned} \Sigma _{\alpha _1,\alpha _2}(z):= \frac{4}{3}\left( (z\cdot \alpha _2)^3 \alpha _1 + (z\cdot \alpha _1)^3\alpha _2\right) . \end{aligned}$$

there $(\alpha _1,\alpha _2)$ is an orthonormal system in ${\mathbb {R}}^2$.

Collecting the results of [12] and [2] we get the following statement:

#### Theorem 1.3

Let $\varepsilon _k \rightarrow 0$ and $u_k \in W^{2,2}_0(\Omega )$ be such that $\limsup _{k\rightarrow \infty } F_{\varepsilon _k}(u_k, \Omega )< \infty$. Then $m_k:=\nabla ^\perp u_k$ is pre-compact in $L^1(\Omega )$. Moreover if $m_k$ converges to *m* in $L^1(\Omega )$, then $|m|=1$ a.e. in $\Omega$, for every entropy $\Phi \in {\mathcal {E}}$ it holds that

$$\begin{aligned} \mu _\Phi := \mathrm {div}\, \Phi ( m) \in {\mathcal {M}} (\Omega ), \end{aligned}$$

where ${\mathcal {M}}(\Omega )$ denotes the set of finite Radon measures on $\Omega$, and

$$\begin{aligned} \left( \bigvee _{(\alpha _1,\alpha _2)} |\mathrm {div}\, \Sigma _{\alpha _1,\alpha _2}(m)|\right) (\Omega )\le \liminf _{k\rightarrow \infty } F_{\varepsilon _k}(u_k,\Omega ), \end{aligned}$$

where $\bigvee$ denotes the supremum operator on non-negative measures (see for example [3, Def. 1.68]).

Theorem 1.3 motivates the introduction of the following space of vector fields, which contains all the limits of sequences $\nabla ^\perp u_{\varepsilon _k}$, where $u_{\varepsilon _k}$ have equi-bounded energy.

#### Definition 1.4

We denote by $A(\Omega )$ the set of all $m\in L^\infty (\Omega ; {\mathbb {R}}^2)$ such that

$$\begin{aligned} \mathrm {div}\, m = 0 \quad \text{ in } {\mathcal {D}}'(\Omega ), \qquad \qquad |m|^2=1 \quad {\mathscr {L}}^2\text{-a.e. } \text{ in } \Omega \end{aligned}$$

and such that for every entropy $\Phi \in {\mathcal {E}}$ it holds that

$$\begin{aligned} \mu _\Phi := \mathrm {div}\left( \Phi (m)\right) \in {\mathcal {M}}_{\text {loc}} (\Omega ), \end{aligned}$$

namely $\mu _\Phi$ is a locally finite Radon measure on $\Omega$. We moreover set

$$\begin{aligned} {\tilde{F}}_0(u,\Omega ):= \left( \bigvee _{(\alpha _1,\alpha _2)} \left| \mathrm {div}{\Sigma _{\alpha _1,\alpha _2}}\left( \nabla ^\perp u\right) \right| \right) (\Omega ). \end{aligned}$$

Finally we denote by

$$\begin{aligned} \Lambda ^0(\Omega ):=\left\{ u \in W^{1,\infty }_0(\Omega ) : \nabla ^\perp u \in A(\Omega )\right\} . \end{aligned}$$

The functional ${\tilde{F}}_0(\cdot ,\Omega )$ coincides with $F_0(\cdot , \Omega )$ in the subspace of $\Lambda ^0(\Omega )$ whose elements have gradient in ${{\,\mathrm{BV}\,}}_{\text {loc}}(\Omega )$ (see [2]) and it is the natural candidate to be the $\Gamma$-limit of the functionals $F_\varepsilon (\cdot , \Omega )$ as $\varepsilon \rightarrow 0^+$.

Although $A(\Omega )\not \subset {{\,\mathrm{BV}\,}}_\text {loc}(\Omega )$, elements of $A(\Omega )$ share with ${{\,\mathrm{BV}\,}}$ functions most of their fine properties.

#### Theorem 1.5

[11] For every $m\in A(\Omega )$ there exists a ${\mathscr {H}}^1$-rectifiable set $J\subset \Omega$ such that

1. for ${\mathscr {H}}^1$-a.e. $x \notin J$ it holds that
  $$\begin{aligned} \lim _{r\rightarrow 0}\frac{1}{r^2}\int _{B_r(x)}|m(y)-{\bar{m}}_{x,r}| dy =0, \end{aligned}$$
  where ${\bar{m}}_{x,r}$ denotes the average of *m* on $B_r(x)$, namely *x* is a vanishing mean oscillation point of *m*;
2. for ${\mathscr {H}}^1$-a.e. $x \in J$ there exist $m^+(x),m^-(x) \in \mathbb S^1$ such that
  $$\begin{aligned} \lim _{r \rightarrow 0} \frac{1}{r^2}\left( \int _{B_r^+(x)} |m(y)-m^+(x)|dy + \int _{B_r^-(x)}|m(y)-m^-(y)|dy \right) =0, \end{aligned}$$
  where $B^\pm (x):= \{ y \in B_r(x): \pm y \cdot {\mathbf {n}}(x)>0\}$ and ${\mathbf {n}}(x)$ is a unit vector normal to *J* in *x*;
3. for every $\Phi \in {\mathcal {E}}$ it holds that
  $$\begin{aligned} \begin{aligned} \mu _\Phi \llcorner J&= [ {\mathbf {n}}\cdot (\Phi (m^+)-\Phi (m^-))] {\mathscr {H}}^1\llcorner J,\\ \mu _\Phi \llcorner K&= 0 \quad \forall K\subset \Omega \setminus J \text{ with } {\mathscr {H}}^1(K)< \infty . \end{aligned} \end{aligned}$$

The analogy with the structure of elements in $A(\Omega )\cap {{\,\mathrm{BV}\,}}_\text {loc}(\Omega )$ is not complete: for these functions properties (1) and (3) can be improved to

1. ${\mathscr {H}}^1$-a.e. $x \notin J$ is a Lebesgue point of *m*;
2. for every $$\Phi \in {\mathcal {E}}$$
  1.6 $$\begin{aligned} \mu _\Phi = [ {\mathbf {n}}\cdot (\Phi (m^+)-\Phi (m^-))] {\mathscr {H}}^1\llcorner J. \end{aligned}$$

In order to prove (3’) from (3) one should show that $\mu _\Phi$ is concentrated on *J*. This is considered as a fundamental step towards the solution of the $\Gamma$-limit conjecture and it remains open. Notice moreover that by means of Theorem 1.5 we can give a meaning to the definition of the functional $F_0(\cdot ,\Omega )$ even for solutions *u* to the eikonal equation with $\nabla ^\perp u\in A(\Omega )\setminus {{\,\mathrm{BV}\,}}_\text {loc}(\Omega )$; Property (3’) would imply that $F_0$ coincides with ${\tilde{F}}_0$ on the whole $\Lambda ^0(\Omega )$.

A fundamental tool in the study of fine properties of elements of $A(\Omega )$ is the kinetic formulation [18] (see also [23] in the framework of scalar conservation laws). Here we use a more recent version obtained in [13].

#### Theorem 1.6

Let $m\in A(\Omega )$. Then there exists $\sigma \in {\mathcal {M}}_\text {loc}(\Omega \times {\mathbb {R}}/2\pi {\mathbb {Z}})$ such that

1.7 $$\begin{aligned} e^{is}\cdot \nabla _x \chi = \partial _s \sigma \qquad \text{ in } {\mathcal {D}}'(\Omega \times {\mathbb {R}}/2\pi {\mathbb {Z}}), \end{aligned}$$

where $\chi : \Omega \times {\mathbb {R}}/2\pi {\mathbb {Z}}$ is defined by

1.8 $$\begin{aligned} \chi (x,s) = {\left\{ \begin{array}{ll} 1 &{}\text{ if } e^{is}\cdot m(x)>0, \\ 0 &{}\text{ otherwise }. \end{array}\right. } \end{aligned}$$

We observe that if $\sigma$ solves (1.7), then

$$\begin{aligned} \sigma + \mu \otimes {\mathcal {L}}^1 \end{aligned}$$

also solves (1.7) for every $\mu \in {\mathcal {M}}_\text {loc}(\Omega )$. This ambiguity is resolved in [13] by considering the unique $\sigma _0$ solving (1.7) such that

$$\begin{aligned} \int _{\Omega \times \mathbb S^1} \varphi (x) d \sigma _0(x,s)=0, \qquad \forall \varphi \in C^\infty _c(\Omega ). \end{aligned}$$

The above kinetic formulation encodes the entropy production of the family of entropies

$$\begin{aligned} {\mathcal {E}}_\pi :=\left\{ \Phi \in {\mathcal {E}}: \frac{d}{ds}\Phi (e^{is})|_{s={\bar{s}}} = - \frac{d}{ds}\Phi (e^{is})|_{s={\bar{s}} + \pi } \right\} . \end{aligned}$$

Condition (1.5) is equivalent to $\frac{d}{ds}\Phi (e^{is}) \cdot e^{is}=0$ for every $s\in {\mathbb {R}}/2\pi {\mathbb {Z}}$, therefore for every $\Phi \in {\mathcal {E}}$ we can define $\psi _\Phi :{\mathbb {R}}/2\pi {\mathbb {Z}}\rightarrow {\mathbb {R}}$ such that

$$\begin{aligned} \frac{d}{ds}\Phi (e^{is}) = 2\psi _\Phi \left( s+ \frac{\pi }{2}\right) e^{i\left( s+\frac{\pi }{2}\right) } \qquad \forall s \in {\mathbb {R}}/2\pi {\mathbb {Z}}. \end{aligned}$$

Notice that $\Phi \in {\mathcal {E}}_\pi$ if and only if $\psi _\Phi$ is $\pi$-periodic. Rephrasing the construction in [13], we have the following identity: for every $\Phi \in {\mathcal {E}}_\pi$ and every $\zeta \in C^1_c (\Omega )$ it holds that

1.9 $$\begin{aligned} \langle \mathrm {div}\Phi (m), \zeta \rangle = \langle \partial _s \sigma , \zeta \otimes \psi _\Phi \rangle , \end{aligned}$$

namely

$$\begin{aligned} \int _\Omega \Phi (m) \cdot \nabla \zeta dx = \int _{\Omega \times {\mathbb {R}}/2\pi {\mathbb {Z}}}\zeta (x) \psi '_\Phi (s) d\sigma . \end{aligned}$$

A possibly weaker version of (3’) is the following:

(3”) Eq. (1.6) holds for every $\Phi \in {\mathcal {E}}_\pi$.

This is equivalent to require that $\nu _0:= (p_x)_\sharp |\sigma _0| \in {\mathcal {M}}_\text {loc}(\Omega )$ is concentrated on *J* and moreover it would be sufficient to establish the equality $F_0={\tilde{F}}_0$. The following proposition is a partial result in this direction for general $m \in A(\Omega )$; we remark here that a key step of the proof of Theorem 1.1 is to establish (3”) for a class of *m* including the limits of $\nabla ^\perp u_\varepsilon$, where $u_\varepsilon$ is a minimizer of $F_\varepsilon (\cdot , \Omega )$ and $\Omega$ is an ellipse.

#### Proposition 1.7

Let $m \in A(\Omega )$ and $(\sigma _{0,x})_{x\in \Omega }\subset {\mathcal {M}}({\mathbb {R}}/2\pi {\mathbb {Z}})$ be the disintegration of $\sigma _0$ with respect to $\nu _0$ defined for $\nu _0$-a.e. $x \in \Omega$ by the properties $|\sigma _{0,x}|({\mathbb {R}}/2\pi {\mathbb {Z}})=1$ and

$$\begin{aligned} \int _{\Omega \times {\mathbb {R}}/2\pi {\mathbb {Z}}}\varphi (x,s) d \sigma _0(x,s) = \int _{\Omega } \int _{{\mathbb {R}}/2\pi {\mathbb {Z}}} \varphi (x,s)d \sigma _{0,x}(s) d \nu _0(x) \end{aligned}$$

for every $\varphi \in C^\infty _c(\Omega \times {\mathbb {R}}/2\pi {\mathbb {Z}})$. Then for $\nu _0$-a.e. $x \in \Omega \setminus J$ there exists ${\bar{s}} = {\bar{s}} (x)\in {\mathbb {R}}/2\pi {\mathbb {Z}}$ such that

$$\begin{aligned} \sigma _{0,x}= \pm \frac{1}{4}\left( \delta _{{\bar{s}}} + \delta _{{\bar{s}} + \pi } - \frac{1}{\pi } {\mathcal {L}}^1\right) . \end{aligned}$$

Among other results, the same expression for $\sigma _{0,x}$ has been obtained very recently in [22] under the additional assumption that $\mathrm {div}\,\Phi (m) \in L^p(\Omega )$ for every $\Phi \in {\mathcal {E}}$. As the authors point out, it is still not known if this additional assumption is sufficient to establish that indeed $\sigma _0$ vanishes.

### The Asymptotic Problem

Adapting the argument in [30] for scalar conservation laws to this context, it is possible to prove that the elements of $A(\Omega )$ with finite energy have strong traces in $L^1$ at the boundary of $\Omega$. However, the conditions

$$\begin{aligned} u_\varepsilon \in \Lambda (\Omega ), \qquad \limsup _{\varepsilon \rightarrow 0} F_{\varepsilon }(u_\varepsilon ,\Omega ) < \infty , \qquad \text{ and } \qquad u=\lim _{\varepsilon \rightarrow 0}u_\varepsilon \quad \text{ in } W^{1,1} \end{aligned}$$

do not guarantee that $\frac{\partial u}{\partial n} =-1$ on $\partial \Omega$; in other words we can have boundary layers. In order to take them into account we slightly reformulate the minimum problem for $F_\varepsilon (\cdot ,\Omega )$: given $\delta >0$ we define

$$\begin{aligned} \Omega _\delta = \{ x \in {\mathbb {R}}^2: {{\,\mathrm{dist}\,}}(x,\Omega ) < \delta \}, \qquad \text{ and } \qquad S_\delta := \Omega _\delta \setminus {\bar{\Omega }}. \end{aligned}$$

Being $\Omega$ of class $C^2$, we can take $\delta >0$ sufficiently small so that the function $- {{\,\mathrm{dist}\,}}(x, \partial \Omega )$ belongs to $W^{2,2}(S_\delta )$. We therefore consider the minimum problems for the functionals $F_\varepsilon (\cdot , \Omega _\delta )$ on the space

$$\begin{aligned} \Lambda _\delta (\Omega ):= \left\{ u\in W^{2,2}(\Omega _\delta ) : u (x)=- {{\,\mathrm{dist}\,}}(x, \partial \Omega ) \text{ for } \text{ a.e. } x \in S_\delta \right\} . \end{aligned}$$

Notice that for every $u \in \Lambda (\Omega )$ the function $u^\delta : \Omega _\delta \rightarrow {\mathbb {R}}$ defined by

1.10 $$\begin{aligned} u^\delta (x):= {\left\{ \begin{array}{ll} u(x) &{} \text{ if } x \in \Omega , \\ - {{\,\mathrm{dist}\,}}(x, \partial \Omega ) &{} \text{ if } x \in \Omega _\delta \setminus \Omega \end{array}\right. } \end{aligned}$$

belongs to $\Lambda _\delta (\Omega )$ and

$$\begin{aligned} F_\varepsilon (u^\delta ,\Omega _\delta ) = F_\varepsilon (u,\Omega ) + \varepsilon \int _{S_\delta } |\nabla ^2 {{\,\mathrm{dist}\,}}(x, \partial \Omega )|^2 dx. \end{aligned}$$

Similarly the restriction to $\Omega$ of any function in $\Lambda _\delta (\Omega )$ belongs to $\Lambda (\Omega )$, so that the two minimum problems are equivalent. We will also denote by

$$\begin{aligned} A_\delta (\Omega ):= \left\{ m \in A(\Omega _\delta ): m = -\nabla ^\perp {{\,\mathrm{dist}\,}}(\cdot , \partial \Omega ) \text{ in } S_\delta \right\} . \end{aligned}$$

We will prove the following result:

#### Theorem 1.8

Let $\Omega$ be an ellipse. Then the function ${\bar{u}}^\delta$, defined by (1.10) with ${\bar{u}} = {{\,\mathrm{dist}\,}}(x, \partial \Omega )$, is the unique minimizer of ${\tilde{F}}_0(\cdot , \Omega _\delta )$ in the space

$$\begin{aligned} \Lambda ^0_\delta (\Omega ):=\left\{ u \in W^{1,2}(\Omega _\delta ) : \nabla ^\perp u \in A(\Omega _\delta ) \text{ and } u= {\bar{u}}^\delta \text{ in } S_\delta \right\} . \end{aligned}$$

We show now that Theorem 1.1 is a corollary of Theorem 1.8 and the previous mentioned results: indeed let $\varepsilon _k\rightarrow 0$ as $k\rightarrow \infty$ and for any *k* let $u_{\varepsilon _k}$ be a minimizer of $F_{\varepsilon _k}(\cdot , \Omega )$ on $\Lambda (\Omega )$. By Theorem 1.3 and (1.3) we have that every limit point $u_0$ of $u_{\varepsilon _k}$ belongs to $\Lambda ^0(\Omega )$ and moreover it holds

$$\begin{aligned} {\tilde{F}}_0(u^\delta _0,\Omega _\delta )\le & {} \liminf _{k\rightarrow \infty } F_{\varepsilon _k}(u^\delta _{\varepsilon _k},\Omega _\delta ) = \liminf _{k\rightarrow \infty } F_{\varepsilon _k}(u_{\varepsilon _k},\Omega )\\= & {} \lim _{k\rightarrow \infty } \min _{\Lambda (\Omega )}F_{\varepsilon _k}(\cdot ,\Omega ) = {\tilde{F}}_0({\bar{u}},\Omega )={\tilde{F}}_0({\bar{u}}^\delta , \Omega _\delta ). \end{aligned}$$

Since ${\bar{u}}^\delta$ is the only minimizer of ${\tilde{F}}_0(\cdot , \Omega _\delta )$ in $\Lambda ^0_\delta (\Omega )$, then $u_0^\delta = {\bar{u}}^\delta$, namely $u_0={\bar{u}}$.

### Related Results

#### Zero-Energy States

The only case in which the behavior of minimizers of $F_\varepsilon (\cdot ,\Omega )$ as $\varepsilon \rightarrow 0$ is completely understood is when $\lim _{\varepsilon \rightarrow 0}\min F_\varepsilon (\cdot ,\Omega )=0$. All the sets $\Omega$ admitting sequences with vanishing energy were characterized in [17] and with the appropriate boundary conditions the limit function is in these cases ${\bar{u}} = {{\,\mathrm{dist}\,}}(\cdot , \partial \Omega )$. A quantitative version of this result is proven in [20] (see also [19]). In a different direction, it was shown in [21] that the vanishing of the two entropy defect measures $\mathrm {div}\Sigma _{e_1,e_2}(m)$ and $\mathrm {div}\Sigma _{\varepsilon _1,\varepsilon _2}(m)$ is sufficient to establish $\mathrm {div}\,\Phi (m)=0$ for every $\Phi \in {\mathcal {E}}$. Here we denoted by $(e_1,e_2)$ the standard orthonormal system in ${\mathbb {R}}^2$ and by

$$\begin{aligned} (\varepsilon _1,\varepsilon _2):= \left( \left( \frac{1}{\sqrt{2}}, \frac{1}{\sqrt{2}} \right) , \left( -\frac{1}{\sqrt{2}}, \frac{1}{\sqrt{2}} \right) \right) \end{aligned}$$

the orthonormal system obtained by performing a rotation of $(e_1,e_2)$ by $\pi /4$.

#### States with a Vanishing Entropy Defect Measure

The case when $\Omega$ is an ellipse is special since we know a priori that there exists an orthonormal system $(\alpha _1,\alpha _2)$ in ${\mathbb {R}}^2$ for which the minimizers $u^\delta$ in $A(\Omega _\delta )$ of the asymptotic problem ${\tilde{F}}_0(\cdot , \Omega _\delta )$ satisfy

1.11 $$\begin{aligned} \mathrm {div}\Sigma _{\alpha _1,\alpha _2}\left( \nabla ^\perp u^\delta \right) =0 \qquad \text{ in } {\mathcal {D}}'(\Omega _\delta ). \end{aligned}$$

This situation has been considered more extensively in [14, 15], where in particular the authors proved the minimizing property of the viscosity solution (1.3) for more general domains and functionals. In this direction we only mention here that the same arguments of this paper allow to prove Theorem 1.1 also in the case where $\Omega$ is a stadium, namely a domain of the form

$$\begin{aligned} \Omega = \{ x \in {\mathbb {R}}^2: {{\,\mathrm{dist}\,}}( x, [0,L]\times \{0\}) < R\} \qquad \text{ for } \text{ some } L,R>0. \end{aligned}$$

We finally mention that under the additional assumption (1.11) we can prove Property (3”).

#### A Micromagnetics Model

A family of functionals $E_\varepsilon$ strictly related to (1.1) was introduced in [28, 29] in the context of micro-magnetics. An analogous result to Theorem 1.1 was proved in [8] even for general smooth domains $\Omega$, while the $\Gamma$-limit conjecture is still open also in this setting. Although Theorem 1.5 has a perfect analogue for the elements in the asymptotic domain of $E_\varepsilon$ (see [7]), the main difficulty seems to be a still not complete understanding of the fine properties of these elements. In this direction we notice that the method used here to establish Proposition 1.7 gives the analogue in this setting of the concentration property (3’) (see [25]).

## Lagrangian Representation of Elements in A${\varvec{(\Omega )}}$

The Lagrangian representation is an extension of the classical method of characteristics to the non-smooth setting: it was introduced in the framework of scalar conservation laws in [9, 24] building on the kinetic formulation from [23]. This approach is strongly inspired by the decomposition in elementary solutions of non-negative measure valued solutions of the linear transport equation, called superposition principle (see [1]). Indeed by Theorem 1.6, the vector fields $m \in A(\Omega )$ are represented by the solution $\chi$ of the linear transport equation (1.7). The main difficulty in this case is due to the source term which is merely a derivative of a measure. This issue is reflected in the lack of regularity of the characteristics detected by our Lagrangian representation, which have bounded variation but they are in general not continuous. A fundamental feature for our analysis is that we can decompose the kinetic measure $\sigma$ in (1.7) along the characteristics.

### Lagrangian Representation

We introduce the following space of curves: given $T>0$, we let

$$\begin{aligned}&\Gamma := \left\{ (\gamma ,t^-_\gamma ,t^+_\gamma ): 0\le t^-_\gamma \le t^+_\gamma \le T, \gamma =(\gamma _x,\gamma _s)\in {{\,\mathrm{BV}\,}}((t^-_\gamma ,t^+_\gamma );\right. \\&\quad \left. \Omega \times {\mathbb {R}}/2\pi {\mathbb {Z}}) , \gamma _x \text{ is } \text{ Lipschitz } \right\} . \end{aligned}$$

We will always consider the right-continuous representative of the component $\gamma _s$. Moreover we will adopt the notation from [3] for the decomposition of the measure *Dv* where $v \in {{\,\mathrm{BV}\,}}(I;{\mathbb {R}})$ for some interval $I\subset {\mathbb {R}}$,

$$\begin{aligned} Dv = {\tilde{D}} v + D^j v, \end{aligned}$$

where ${\tilde{D}} v$ denotes the sum of the absolutely continuous part and the Cantor part of *Dv* and $D^jv$ denotes the jump part of *Dv*. We will need to consider also ${\tilde{D}} v$ for functions $v\in {{\,\mathrm{BV}\,}}(I;{\mathbb {R}}/2\pi {\mathbb {Z}})$. In this case ${\tilde{D}}v = {\tilde{D}}w$ where *w* is any function in ${{\,\mathrm{BV}\,}}(I;{\mathbb {R}})$ such that for every $z \in I$ the value *w*(*z*) belongs to the class *v*(*z*) in ${\mathbb {R}}/2\pi {\mathbb {Z}}$. For every $t \in (0,T)$ we consider the section

$$\begin{aligned} \Gamma (t):= \left\{ \left( \gamma ,t^-_\gamma ,t^+_\gamma \right) \in \Gamma : t \in \left( t^-_\gamma ,t^+_\gamma \right) \right\} , \end{aligned}$$

and we denote

$$\begin{aligned} \begin{aligned} e_t: \Gamma (t)&\rightarrow \Omega \times {\mathbb {R}}/2\pi {\mathbb {Z}}\\ (\gamma , t^-_\gamma , t^+_\gamma )&\mapsto \gamma (t). \end{aligned} \end{aligned}$$

#### Definition 2.1

Let $m\in A(\Omega )$ and $\Omega '$ be a $W^{2,\infty }$-open set compactly contained in $\Omega$ We say that a finite non-negative Radon measure $\omega \in {\mathcal {M}}( \Gamma )$ is a *Lagrangian representation* of *m* in $\Omega '$ if the following conditions hold:

1. for every $t\in (0,T)$ it holds that
  2.1 $$\begin{aligned} ( e_t)_\sharp \left[ \omega \llcorner \Gamma (t)\right] = \chi {\mathscr {L}}^{2}\times {\mathscr {L}}^1, \end{aligned}$$
  where $\chi$ is defined in (1.8);
2. the measure $\omega$ is concentrated on curves $(\gamma ,t^-_\gamma ,t^+_\gamma )\in \Gamma$ such that for ${\mathscr {L}}^1$-a.e. $t \in (t^-_\gamma ,t^+_\gamma )$ the following characteristic equation holds:
  2.2 $$\begin{aligned} {\dot{\gamma }}_x(t)= e^{i \gamma _s(t)}; \end{aligned}$$
3. it holds the integral bound
  $$\begin{aligned} \int _{ \Gamma } \text {Tot.Var.}_{(0,T)} \gamma _s d\omega (\gamma ) <\infty ; \end{aligned}$$
4. for $\omega$-a.e. $(\gamma ,t^-_\gamma ,t^+_\gamma )\in \Gamma$ it holds that
  $$\begin{aligned} t^-_\gamma >0 \Rightarrow \gamma _x(t^-_\gamma +) \in \partial \Omega ', \qquad \text{ and } \qquad t^+_\gamma <T \Rightarrow \gamma _x(t^+_\gamma -) \in \partial \Omega '. \end{aligned}$$

For every curve $\gamma \in \Gamma$ we define the measure $\sigma _\gamma \in {\mathcal {M}}((0,T)\times \Omega ' \times {\mathbb {R}}/2\pi {\mathbb {Z}})$ by

2.3 $$\begin{aligned} \sigma _\gamma =(\mathrm {id}, \gamma )_\sharp {\tilde{D}}_t \gamma _s + {\mathscr {H}}^1\llcorner E_{\gamma }^+ -{\mathscr {H}}^1\llcorner E_{\gamma }^-, \end{aligned}$$

where

2.4 $$\begin{aligned} \begin{aligned} E_\gamma ^+&:= \{(t,x,s) \in (0,T)\times \Omega \times {\mathbb {R}}/2\pi {\mathbb {Z}}: \gamma _x(t)=x \text{ and } \gamma _s(t-)\\&\le s \le \gamma _s(t+)\le \gamma _s(t-)+\pi \}, \\ E_\gamma ^-&:= \{(t,x,s) \in (0,T)\times \Omega \times {\mathbb {R}}/2\pi {\mathbb {Z}}: \gamma _x(t)=x \text{ and } \gamma _s(t+)\\&\le s \le \gamma _s(t-)< \gamma _s(t+)+\pi \}. \end{aligned} \end{aligned}$$

Notice that since ${\mathbb {R}}/2\pi {\mathbb {Z}}$ is not ordered, given $s_1\ne s_2 \in {\mathbb {R}}/2\pi {\mathbb {Z}}$ the condition $s_1< s_2$ is not defined. Nevertheless we use the notation $s \in (s_1,s_2)$ or $s_1<s<s_2$ to indicate the following condition (depending only on the orientation of ${\mathbb {R}}/2\pi {\mathbb {Z}}$): if $t_1,t_2 \in {\mathbb {R}}$ are such $t_1<t_2<t_1+2\pi$, $e^{it_1}=e^{is_1}$ and $e^{it_2}=e^{is_2}$ then there exists $t \in (t_1,t_2)$ such that $e^{it}=e^{is}$.

#### Lemma 2.2

Let $\omega$ be a Lagrangian representation of $m\in A(\Omega )$ on $\Omega '$. Let us denote by

$$\begin{aligned} \sigma _\omega := - \int _\Gamma \sigma _\gamma d\omega \end{aligned}$$

and by ${\tilde{\chi }} :(0,T)\times \Omega \times {\mathbb {R}}/2\pi {\mathbb {Z}}\rightarrow {\mathbb {R}}$ the function defined by ${\tilde{\chi }}(t,x,s)=\chi (x,s)$ for every $t \in (0,T)$. Then it holds that

2.5 $$\begin{aligned} e^{is}\cdot \nabla _x {\tilde{\chi }} = \partial _s \sigma _\omega \quad \in {\mathcal {D}}'((0,T)\times \Omega '\times {\mathbb {R}}/2\pi {\mathbb {Z}}). \end{aligned}$$

#### Proof

We show that (2.5) holds when tested with every function of the form $\phi (t,x,s)=\zeta (t)\varphi (x,s)$ with $\zeta \in C^\infty _c((0,T))$ and $\varphi \in C^\infty _c(\Omega '\times {\mathbb {R}}/2\pi {\mathbb {Z}})$. It follows from (2.1) and (2.2) that

2.6 $$\begin{aligned} \begin{aligned} \langle e^{is}\cdot \nabla _x {\tilde{\chi }}, \phi \rangle =&~ - \int e^{is}\cdot \nabla _x \varphi (x,s) \zeta (t) {\tilde{\chi }}(t,x,s)dtdxds \\ =&~- \int _{(0,T)}\int _{\Gamma (t)} e^{i\gamma _s(t)}\cdot \nabla _x \varphi ( \gamma (t)) d\omega \zeta (t) dt \\ =&~- \int _\Gamma \int _{t^-_\gamma }^{t^+_\gamma } \frac{d}{dt} \gamma _x(t) \cdot \nabla _x \varphi (\gamma (t)) \zeta (t) dt d\omega . \end{aligned} \end{aligned}$$

By the chain-rule for functions with bounded variation we have the following equality between measures:

$$\begin{aligned} \frac{d}{dt}\varphi \circ \gamma= & {} \nabla _x \varphi (\gamma (t)) \cdot \frac{d}{dt} \gamma _x(t) + \partial _s\varphi (\gamma (t)) {\tilde{D}}_t \gamma _s + \sum _{t_j \in J_\gamma } \left( \varphi (t_j,\gamma (t_j+)) \right. \\&\left. - \varphi (t_j,\gamma (t_j-))\right) \delta _{t_j}, \end{aligned}$$

where $J_{\gamma }$ denotes the jump set of $\gamma$. Therefore, proceeding in the chain (2.6), we have

$$\begin{aligned} \begin{aligned} \langle e^{is}\cdot \nabla _x {\tilde{\chi }}, \phi \rangle =&~ - \int _\Gamma \int _{t^-_\gamma }^{t^+_\gamma } \frac{d}{dt} \varphi (\gamma (t)) \zeta (t) dt d\omega + \int _\Gamma \int _{t^-_\gamma }^{t^+_\gamma } \partial _s\varphi (\gamma (t))\zeta (t) d {\tilde{D}}_t \gamma _s(t) d\omega \\&~ + \int _\Gamma \sum _{t_j \in J_\gamma } \big ( \varphi (t_j,\gamma (t_j+)) - \varphi (t_j,\gamma (t_j-))\big ) \zeta (t_j) d\omega . \end{aligned} \end{aligned}$$

By definition of $\sigma _\gamma$ in (2.3) it holds that

$$\begin{aligned}&\int _{t^-_\gamma }^{t^+_\gamma } \partial _s\varphi (\gamma (t))\zeta (t) d {\tilde{D}}_t \gamma _s(t) + \sum _{t_j \in J_\gamma } \big ( \varphi (t_j,\gamma (t_j+)) - \varphi (t_j,\gamma (t_j-))\big ) \zeta (t_j) d\omega \\&\quad = \int \partial _s \varphi (x,s) \zeta (t) d \sigma _\gamma , \end{aligned}$$

therefore in order to establish $\langle e^{is}\cdot \nabla _x {\tilde{\chi }}, \phi \rangle = \langle \partial _s \sigma _\omega , \phi \rangle$ it suffices to prove that

$$\begin{aligned} \int _\Gamma \int _{t^-_\gamma }^{t^+_\gamma } \frac{d}{dt} \varphi (\gamma (t)) \zeta (t) dt d\omega = 0. \end{aligned}$$

By Point (4) in Definition 2.1 for $\omega$-a.e. $\gamma \in \Gamma$ it holds that $\varphi (\gamma (t^-_\gamma +))= \varphi (\gamma (t^+_\gamma -))=0$, and, in particular,

$$\begin{aligned} \begin{aligned} \int _\Gamma \int _{t^-_\gamma }^{t^+_\gamma } \frac{d}{dt} \varphi (\gamma (t)) \zeta (t) dt d\omega =&~ -\int _\Gamma \int _{t^-_\gamma }^{t^+_\gamma }\varphi (\gamma (t)) \zeta '(t) dt d\omega \\ =&~ \int _{(0,T)\times \Omega \times {\mathbb {R}}/2\pi {\mathbb {Z}}} {\tilde{\chi }} \varphi (x,s)\zeta '(t) dt dx ds \\ =&~ 0, \end{aligned} \end{aligned}$$

where we used (2.1) in the second equality and that ${\tilde{\chi }}$ does not depend on *t* in the last equality. This concludes the proof. $\square$

#### Definition 2.3

We say that $\sigma \in {\mathcal {M}}_{\text {loc}}(\Omega '\times {\mathbb {R}}/2\pi {\mathbb {Z}})$ is a *minimal kinetic measure* if it satisfies (1.7) and for every $\sigma '$ solving (1.7) it holds that

$$\begin{aligned} \nu _\sigma := (p_x)_\sharp |\sigma | \le (p_x)_\sharp |\sigma '| =: \nu _{\sigma '}. \end{aligned}$$

We moreover say that $\omega$ is a a *minimal Lagrangian representation* of *m* if it is a Lagrangian representation of *m* according to Def. 2.1 and

$$\begin{aligned} \sigma _\omega = {\mathscr {L}}^1 \otimes \sigma _t \end{aligned}$$

with $\sigma _t$ minimal kinetic measure for ${\mathscr {L}}^1$-a.e. $t\in (0,T)$.

The existence of a minimal kinetic measure is proven in the following lemma:

#### Lemma 2.4

For every $m\in A(\Omega )$ there exists a minimal kinetic measure $\sigma$. Moreover there exists $\nu _{\min }\in {\mathcal {M}}_{\text {loc}}(\Omega )$ such that for every minimal kinetic measure $\sigma$ it holds that $\nu _{\min } = (p_x)_\sharp |\sigma |$.

#### Proof

Since $\partial _s \sigma$ is uniquely determined by (1.7), we have that a kinetic measure $\sigma$ is minimal if and only if for $\nu _\sigma$-a.e. $x \in \Omega$ the disintegration $\sigma _x$ satisfies the following inequality:

2.7 $$\begin{aligned} 1= \Vert \sigma _x \Vert \le \left\| \sigma _x + \alpha {\mathscr {L}}^1\right\| \qquad \forall \alpha \in {\mathbb {R}}. \end{aligned}$$

Therefore all minimal kinetic measures are of the form

$$\begin{aligned} \nu _{\sigma _0} \otimes \left( (\sigma _0)_x + \alpha (x) {\mathcal {L}}^1 \right) , \end{aligned}$$

where $\alpha : \Omega \rightarrow {\mathbb {R}}$ is a measurable function such that for $\nu _{\sigma _0}$-a.e. $x \in \Omega$ it holds that

2.8 $$\begin{aligned} \left\| (\sigma _0)_x + \alpha (x) {\mathcal {L}}^1 \right\| \le \left\| (\sigma _0)_x + c{\mathcal {L}}^1\right\| \qquad \forall c \in {\mathbb {R}}. \end{aligned}$$

The existence of such an $\alpha$ is trivial and in particular it holds that

$$\begin{aligned} \nu _{\min } = \left( \min _{\alpha \in {\mathbb {R}}} \left\| (\sigma _0)_x + \alpha {\mathcal {L}}^1 \right\| \right) \nu _0. \end{aligned}$$

$$\square $$

In Sect. 3 we will show that for every $m\in A(\Omega )$ there exists a *unique* minimal kinetic measure $\sigma _{\min }$, namely that for $\nu _{\min }$-a.e. $x \in \Omega$ there exists a unique $\alpha (x)$ such that (2.8) holds.

The main result of this section is

#### Proposition 2.5

Let $\Omega \subset {\mathbb {R}}^2$ be a bounded open set, $m\in A(\Omega )$ and $\Omega '$ be a $W^{2,\infty }$ open set compactly contained in $\Omega$ be such that ${\mathscr {H}}^1$-a.e. $x \in \partial \Omega '$ is a Lebesgue point of *m*. Then there exists a minimal Lagrangian representation $\omega$ of *m* on $\Omega '$. In particular it holds that

2.9 $$\begin{aligned} |\sigma _\omega |=\int _\Gamma |\sigma _\gamma |d\omega . \end{aligned}$$

The existence of a Lagrangian representation for weak solutions with finite entropy production to general conservation laws on the whole $(0,T)\times {\mathbb {R}}^d$ has been proved in [24]. The case of bounded domains when $\Omega '$ is a ball was considered in [25] for the class of solutions to the eikonal equation arising in [29]. The extension to the case where $\Omega '$ is a $W^{2,\infty }$ open set does not cause any significant difficulty. In particular the argument proposed in [25] applies here with trivial modifications and leads to the following partial result:

#### Lemma 2.6

In the setting of Proposition 2.5, let $\sigma \in {\mathcal {M}}_{\text {loc}}(\Omega \times {\mathbb {R}}/2\pi {\mathbb {Z}})$ be a locally finite measure satisfying (1.7). Then there exists a Lagrangian representation $\omega$ of *m* on $\Omega '$ such that

$$\begin{aligned} \int _\Gamma \text {Tot.Var.}_{(t^-_\gamma ,t^+_\gamma )}\gamma _s d\omega \le T |\sigma |(\Omega ' \times {\mathbb {R}}/2\pi {\mathbb {Z}}). \end{aligned}$$

We now prove Proposition 2.5 relying on Lemma 2.6.

#### Proof of Proposition 2.5

Let $m\in A(\Omega )$ and let ${\bar{\sigma }}$ be a minimal kinetic measure. By Lemma 2.6, there exists a Lagrangian representation $\omega$ of *m* such that

2.10 $$\begin{aligned} \int _\Gamma \text {Tot.Var.}_{(t^-_\gamma ,t^+_\gamma )}\gamma _s d\omega \le T \Vert {\bar{\sigma }}\Vert . \end{aligned}$$

By definition of $\sigma _\omega$ it holds that

2.11 $$\begin{aligned} \Vert \sigma _\omega \Vert \le \left( \int _\Gamma |\sigma _\gamma |d\omega \right) \big ((0,T)\times \Omega \times {\mathbb {R}}/2\pi {\mathbb {Z}}\big ) = \int _\Gamma \text {Tot.Var.}_{(t^-_\gamma ,t^+_\gamma )}\gamma _s d\omega .\nonumber \\ \end{aligned}$$

By Lemma 2.2, the measure $\sigma _\omega$ satisfies (2.5); being ${\bar{\sigma }}$ a minimal kinetic measure for *m*, it follows that $T\Vert {\bar{\sigma }}\Vert \le \Vert \sigma _\omega \Vert$. In particular the inequalities in (2.10) and (2.11) are equalities and (2.9) follows. $\square$

The following lemma is a simple application of Tonelli theorem and (2.1); since it is already proven in [26], we refer to it for the details.

#### Lemma 2.7

For $\omega$-a.e. $(\gamma ,t^-_\gamma ,t^+_\gamma ) \in \Gamma$ it holds that for ${\mathscr {L}}^1$-a.e. $t\in (t^-_\gamma ,t^+_\gamma )$

1. $\gamma _x(t)$ is a Lebesgue point of *m*;
2. $$e^{i\gamma _s(t)} \cdot m(\gamma _x(t))>0$$.

We denote by $\Gamma _g$ the set of curves $\gamma \in \Gamma$ such that the two properties above hold.

## Structure of the Kinetic Measure

The main goal of this section is to prove Proposition 1.7. As a corollary we will obtain the concentration property (3”) presented in the introduction for solutions $m\in A(\Omega )$ with a vanishing entropy defect measure. The key step is the following regularity result (the strategy of the proof is borrowed from [25], where an analogous statement was proved for the solutions to the eikonal equation arising in the micromagnetics model mentioned in the introduction, and we finally observe that in that situation this result is sufficient to establish the concentration property (3’), while it is not the case here):

### Lemma 3.1

Let ${\bar{\gamma }} \in \Gamma _g$ and ${\bar{t}} \in (t^-_{{\bar{\gamma }}},t^+_{{\bar{\gamma }}})$, and set ${\bar{x}}:={\bar{\gamma }}_x({\bar{t}})$ and ${\bar{s}}:= {\bar{\gamma }}_s({\bar{t}}+)$. Then there exists $c>0$ such that for every $\delta \in (0,1/2)$ we have at least one of the following:

1. the lower density estimate holds true:
  $$\begin{aligned} \liminf _{r\rightarrow 0} \frac{{\mathscr {L}}^2\left( \left\{ x \in B_r({\bar{x}}): e^{i {\bar{s}}}\cdot m(x) > -\delta \right\} \right) }{r^2} \ge c\delta ; \end{aligned}$$
2. the following lower bound holds true:
  $$\begin{aligned} \limsup _{r\rightarrow 0}\frac{\nu _{\min }(B_r({\bar{x}}))}{r}\ge c\delta ^3. \end{aligned}$$

The same statement holds by setting ${\bar{s}} := {\bar{\gamma }}_s({\bar{t}}-)$.

### Proof

We prove the lemma only for ${\bar{s}}= {\bar{\gamma }}_s({\bar{t}}+)$, being the case ${\bar{s}} = {\bar{\gamma }}_s({\bar{t}}-)$ analogous. Let $\delta _1>0$ be sufficiently small so that for ${\mathscr {L}}^1$-a.e. $t\in ({\bar{t}},{\bar{t}} + \delta _1)$ it holds that

3.1 $$\begin{aligned} e^{i {\bar{\gamma }}_s(t)}\cdot e^{i{\bar{s}}}\ge \cos \left( \frac{\delta }{5}\right) . \end{aligned}$$

Since ${\bar{\gamma }}_x$ satisfies (2.2), then for every $r \in \left( 0,\frac{\delta _1}{2}\right)$ there exists $t_r \in ({\bar{t}}, {\bar{t}} + \delta _1)$ such that

$$\begin{aligned} {\bar{\gamma }}_x(t) \in B_r({\bar{x}}) \quad \forall t \in ({\bar{t}}, t_r), \qquad \text{ and } \qquad {\bar{\gamma }}_x(t_r)\in \partial B_r({\bar{x}}). \end{aligned}$$

Moreover since $\cos (\delta /5) \in (1/2,1)$, then (3.1) implies

$$\begin{aligned} r\le t_r -{\bar{t}} \le 2r. \end{aligned}$$

For every $r \in \left( 0,\frac{\delta _1}{2}\right)$ we denote by

$$\begin{aligned} \begin{aligned} E_+(r)&:= \{ t \in ({\bar{t}},t_r) : m({\bar{\gamma }}_x(t))\cdot e^{i({\bar{\gamma }}_s(t)+\delta )}>0\}, \\ E_-(r)&:= \{ t \in ({\bar{t}},t_r) : m({\bar{\gamma }}_x(t))\cdot e^{i({\bar{\gamma }}_s(t)-\delta )}>0\}. \end{aligned} \end{aligned}$$

Since $\gamma \in \Gamma _g$, for ${\mathscr {L}}^1$-a.e. $t\in (0,t_r)$ it holds that

$$\begin{aligned} m({\bar{\gamma }}_x(t))\cdot e^{i{\bar{\gamma }}_s(t)}>0, \end{aligned}$$

therefore, being $\delta \in \left( 0,\frac{1}{2}\right)$, we have

$$\begin{aligned} ({\bar{t}}, t_r)\subset E_+(r)\cup E_-(r). \end{aligned}$$

In particular

$$\begin{aligned} {\mathscr {L}}^1(E_+(r)) + {\mathscr {L}}^1(E_-(r)) \ge t_r -{\bar{t}}\ge r. \end{aligned}$$

In the remaining part of the proof we assume that ${\mathscr {L}}^1(E_-(r))>r/2$, being the case ${\mathscr {L}}^1(E_+(r))>r/2$ analogous.

Given $\varepsilon >0$, we consider the strip

3.2 $$\begin{aligned} S_{r,\varepsilon }:=\left\{ x \in \Omega _\delta : \exists t \in ({\bar{t}}, t_r): \left| {\bar{\gamma }}_x(t)-x\right| <\varepsilon \right\} . \end{aligned}$$

For every $(\gamma ,t^-_\gamma ,t^+_\gamma ) \in \Gamma$ let $(t^-_{\gamma ,i},t^+_{\gamma ,i})_{i=1}^{N_{\gamma }}$ be the nontrivial interiors of the connected components of $\gamma _s^{-1}\left( \left( {\bar{s}} - \delta \right) , {\bar{s}} - \frac{2}{5}\delta \right)$ which intersect $\gamma ^{-1}\left( S_{r,\varepsilon }\times \left( {\bar{s}} - \frac{4}{5}\delta , {\bar{s}} - \frac{3}{5}\delta \right) \right)$. Notice that we have the estimate

$$\begin{aligned} N_\gamma \le 1 + \frac{5}{\delta }\text {Tot.Var.}\gamma _s. \end{aligned}$$

For every $i\in {\mathbb {N}}$ we consider

$$\begin{aligned} \Gamma _i:= \{(\gamma , t^-_\gamma ,t^+_\gamma )\in \Gamma : N_{\gamma }\ge i\} \end{aligned}$$

and the measurable restriction map

$$\begin{aligned} \begin{aligned} R_{i}: \Gamma _{i}&\rightarrow \Gamma . \\ (\gamma ,t^-_\gamma ,t^+_\gamma )&\mapsto (\gamma , t^-_{\gamma ,i},t^+_{\gamma ,i}) \end{aligned} \end{aligned}$$

We finally consider the measure

$$\begin{aligned} {\tilde{\omega }}:= \sum _{i=1}^\infty (R_{i})_\sharp \left( \omega \llcorner \Gamma _i\right) . \end{aligned}$$

We observe that ${\tilde{\omega }}\in {\mathcal {M}}_+( \Gamma )$, since, for every $N>0$,

$$\begin{aligned} \left\| \sum _{i=1}^N (R_{i})_\sharp \left( \omega \llcorner \Gamma _i\right) \right\| \le \int _{ \Gamma } N_{\gamma } d\omega \le \int _{ \Gamma } \left( 1 + \frac{5}{\delta }\text {Tot.Var.}\gamma _s\right) d\omega (\gamma ) < \infty , \end{aligned}$$

by Point (3) in Definition 2.1. The advantage of the measure ${\tilde{\omega }}$ is that it is concentrated on curves whose *x*-components are transversal to ${\bar{\gamma }}_x$ on the whole domain of definition. This property allows us to prove the following claim:

### Claim 1

There exists an absolute constant ${\tilde{c}}>0$ such that for ${\tilde{\omega }}$-a.e. $(\gamma ,t^-_\gamma ,t^+_\gamma )\in \Gamma$ it holds

$$\begin{aligned} {\mathscr {L}}^1\left( \left\{ t \in (t^-_\gamma ,t^+_\gamma ) : \gamma (t) \in S_{r,\varepsilon }\times \left( {\bar{s}} - \frac{4}{5}\delta , {\bar{s}} -\frac{3}{5}\delta \right) \right\} \right) \le {\tilde{c}} \frac{\varepsilon }{\delta }. \end{aligned}$$

### Proof of Claim 1

It follows from (3.1) and the characteristic equation (2.2) that there exists a Lipschitz function $f_{{\bar{\gamma }}}:{\mathbb {R}}\rightarrow {\mathbb {R}}$ such that

3.3 $$\begin{aligned}&\big \{{\bar{\gamma }}_x(t):t\in ({\bar{t}}, {\bar{t}} + \delta _1)\big \} \subset \left\{ z e^{i{\bar{s}}} + f_{{\bar{\gamma }}}(z) e^{i\left( {\bar{s}} + \frac{\pi }{2}\right) }: z \in {\mathbb {R}}\right\} \qquad \text{ and }\nonumber \\&\quad {{\,\mathrm{Lip}\,}}(f_{{\bar{\gamma }}})\le \tan \left( \frac{\delta }{5}\right) . \end{aligned}$$

Similarly for ${\tilde{\omega }}$-a.e. $(\gamma ,t^-_\gamma ,t^+_\gamma ) \in \Gamma$ there exists a Lipschitz function $f_\gamma$ such that

$$\begin{aligned}&\big \{\gamma _x(t):t\in (t^-_\gamma , t^+_\gamma )\big \} \subset \left\{ z e^{i{\bar{s}}} + f_{\gamma }(z) e^{i\left( {\bar{s}} + \frac{\pi }{2}\right) }: z \in {\mathbb {R}}\right\} \qquad \text{ and }\\&\quad \frac{d}{dz}f_\gamma (z) \in \left( -\tan \delta , -\tan \left( \frac{2}{5}\delta \right) \right) \end{aligned}$$

for ${\mathscr {L}}^1$-a.e. $z \in {\mathbb {R}}$. By the definitions of $S_{r,\varepsilon }$ in (3.2) and of $f_{{\bar{\gamma }}}$ in (3.3), it easily follows that

3.4 $$\begin{aligned} S_{r,\varepsilon }\subset \left\{ x \in \Omega _\delta : f_{{\bar{\gamma }}} \left( x\cdot e^{i{\bar{s}}}\right) - \varepsilon \left( \cos \left( \frac{\delta }{5}\right) \right) ^{-1}\right.\le & {} x\cdot e^{i\left( {\bar{s}} + \frac{\pi }{2}\right) } \le f_{{\bar{\gamma }}} \left( x\cdot e^{i{\bar{s}}}\right) \nonumber \\&\left. +\,\varepsilon \left( \cos \left( \frac{\delta }{5}\right) \right) ^{-1} \right\} . \end{aligned}$$

Given $(\gamma ,t^-_\gamma ,t^+_\gamma )\in \Gamma$ let us consider the function $g_\gamma :(t^-_\gamma ,t^+_\gamma )\rightarrow {\mathbb {R}}$ defined by

$$\begin{aligned} g_\gamma (t)= \gamma _x(t)\cdot e^{i\left( {\bar{s}} + \frac{\pi }{2}\right) }. \end{aligned}$$

By construction of ${\tilde{\omega }}$, for ${\tilde{\omega }}$-a.e. $(\gamma ,t^-_\gamma ,t^+_\gamma )\in \Gamma$ and ${\mathscr {L}}^1$-a.e. $t \in (t^-_\gamma ,t^+_\gamma )$ it holds that

3.5 $$\begin{aligned} \frac{d}{dt}g_\gamma (t) \le - \sin \left( \frac{2}{5}\delta \right) . \end{aligned}$$

On the other hand

3.6 $$\begin{aligned} \frac{d}{dt}f_{{\bar{\gamma }}}(\gamma _x(t)\cdot e^{i{\bar{s}}}) \ge - \sin \left( \frac{\delta }{5}\right) . \end{aligned}$$

By (3.4), for every $t\in (t^-_\gamma ,t^+_\gamma )$ such that $\gamma _x(t) \in S_{r,\varepsilon }$ it holds that

$$\begin{aligned} f_{{\bar{\gamma }}} (\gamma _x(t)\cdot e^{i{\bar{s}}}) - \varepsilon \left( \cos \left( \frac{\delta }{5}\right) \right) ^{-1} \le g_\gamma (t) \le f_{{\bar{\gamma }}} (\gamma _x(t)\cdot e^{i{\bar{s}}}) + \varepsilon \left( \cos \left( \frac{\delta }{5}\right) \right) ^{-1}. \end{aligned}$$

Therefore, by (3.5) and (3.6), we have

$$\begin{aligned} {\mathscr {L}}^1\left( \{t: \gamma _x(t) \in S_{r,\varepsilon }\} \right) \le \frac{2\varepsilon \left( \cos \left( \frac{\delta }{5}\right) \right) ^{-1} }{\left| \sin \left( \frac{2}{5}\delta \right) - \sin \left( \frac{\delta }{5}\right) \right| } \le {\tilde{c}}\frac{\varepsilon }{\delta }, \end{aligned}$$

for some universal ${\tilde{c}}>0$. This concludes the proof of the claim. $\square$

By construction we have

$$\begin{aligned} (e_t)_\sharp {\tilde{\omega }} \ge {\mathscr {L}}^3 \llcorner \left\{ (x,s) \in S_{r,\varepsilon }\times \left( {\bar{s}} - \frac{4}{5}\delta , {\bar{s}} - \frac{3}{5}\delta \right) : m(x)\cdot e^{i s}>0\right\} \end{aligned}$$

for every $t \in (0,T)$. Therefore

3.7 $$\begin{aligned} \begin{aligned} T {\mathscr {L}}^3&~\left( \left\{ (x,s) \in S_{r,\varepsilon }\times \left( {\bar{s}} - \frac{4}{5}\delta , {\bar{s}} - \frac{3}{5}\delta \right) : m(x)\cdot e^{i s}>0\right\} \right) \\&~\le \int _{\Gamma }{\mathscr {L}}^1 \left( \left\{ t: \gamma (t) \in S_{r,\varepsilon }\times \left( {\bar{s}} - \frac{4}{5}\delta , {\bar{s}} - \frac{3}{5}\delta \right) \right\} \right) d{\tilde{\omega }} \\&~ \le {\tilde{c}} \frac{\varepsilon }{\delta }{\tilde{\omega }} (\Gamma ). \end{aligned} \end{aligned}$$

On the other hand, since ${\bar{\gamma }}\in \Gamma _g$ and ${\mathscr {L}}^1(E_-(r))>r/2$ there exists ${\bar{\varepsilon }}>0$ such that for every $\varepsilon \in (0,{\bar{\varepsilon }})$ it holds that

3.8 $$\begin{aligned} \begin{aligned}&{\mathscr {L}}^3 \left( \left\{ (x,s) \in S_{r,\varepsilon }\times \left( {\bar{s}} - \frac{4}{5}\delta , {\bar{s}} - \frac{3}{5}\delta \right) : m(x)\cdot e^{i s}>0\right\} \right) \\&\quad \ge ~ \frac{1}{2} {\mathscr {L}}^3 \left( S_{r,\varepsilon }\times \left( {\bar{s}} - \frac{4}{5}\delta , {\bar{s}} - \frac{3}{5}\delta \right) \right) \\&\quad \ge ~ \frac{\varepsilon r \delta }{5}. \end{aligned} \end{aligned}$$

By (3.7) and (3.8) it follows that

$$\begin{aligned} {\tilde{\omega }} (\Gamma ) \ge \frac{\varepsilon r \delta }{5} \cdot \frac{\delta T }{{\tilde{c}} \varepsilon } = \frac{r\delta ^2}{5 {\tilde{c}}}T. \end{aligned}$$

We consider the split $\Gamma = \Gamma _> \cup \Gamma _<$, where

$$\begin{aligned}&\Gamma _>:=\{(\gamma ,t^-_\gamma ,t^+_\gamma ) \in \Gamma : t^+_\gamma - t^-_\gamma \ge r \}, \quad \text{ and } \\&\Gamma _<:=\{(\gamma ,t^-_\gamma ,t^+_\gamma ) \in \Gamma : t^+_\gamma - t^-_\gamma < r \}. \end{aligned}$$

We will prove the following claim, from which the lemma follows immediately:

### Claim 2

There exists an absolute constant $c_1>0$ such that the two following implications hold true:

1. if $${\tilde{\omega }} (\Gamma _>)\ge \frac{r\delta ^2T}{10 {\tilde{c}}}$$, then
  $$\begin{aligned} {\mathscr {L}}^2\left( \left\{ x \in B_{2r} ({\bar{x}}) : e^{i{\bar{\gamma }}_s({\bar{t}}+)}\cdot m(x)>-\delta \right\} \right) \ge c_1\delta r^2; \end{aligned}$$
2. if $${\tilde{\omega }} (\Gamma _<)\ge \frac{r\delta ^2T}{10 {\tilde{c}}}$$, then
  $$\begin{aligned} \nu (B_{2r}({\bar{x}})) \ge c_1 \delta ^3 r. \end{aligned}$$

*Proof of (1)*. By definition of $\Gamma _>$ and the assumption in (1) we have

$$\begin{aligned} \begin{aligned} T \frac{r^2\delta ^2}{10 {\tilde{c}}} \le&~ \int _{\Gamma }{\mathscr {L}}^1\left( \left\{ t \in (t^-_\gamma ,t^+_\gamma ): \gamma (t) \in B_{2r}({\bar{x}})\times \left( {\bar{s}} - \delta , {\bar{s}} - \frac{2}{5}\delta \right) \right\} \right) d{\tilde{\omega }} \\ \le&~T {\mathscr {L}}^3\left( \left\{ (x,s) \in B_{2r}({\bar{x}}) \times \left( {\bar{s}} - \delta , {\bar{s}} - \frac{2}{5}\delta \right) : m(x)\cdot e^{is}> 0 \right\} \right) \\ \le&~T\delta {\mathscr {L}}^2\left( \left\{ x \in B_{2r}({\bar{x}}) : m(x)\cdot e^{i {\bar{s}}}>-\delta \right\} \right) . \end{aligned} \end{aligned}$$

*Proof of (2)*. For ${\tilde{\omega }}$-a.e. $(\gamma ,t^-_\gamma ,t^+_\gamma )\in \Gamma _<$, the image of $\gamma _x$ is contained in $B_{2r}({\bar{x}})$ and $\text {Tot.Var.}(\gamma _s)\ge \frac{\delta }{5}$. Since $\omega$ is a minimal Lagrangian representation, this implies that

$$\begin{aligned} T\nu _{\min }(B_{2r}({\bar{x}})) = |\sigma _\omega |((0,T)\times B_{2r}({\bar{x}})) \ge \int _{\Gamma _<}\text {Tot.Var.}\gamma _s d{\tilde{\omega }} \ge \frac{\delta }{5}{\tilde{\omega }}(\Gamma _<) \ge \frac{Tr\delta ^3}{50{\tilde{c}}}. \end{aligned}$$

$$\square $$

### Proposition 3.2

Let $m\in A(\Omega )$ and $\sigma \in {\mathcal {M}}(\Omega \times {\mathbb {R}}/2\pi {\mathbb {Z}})$ be a minimal kinetic measure. Then for $\nu _{\min }$-a.e. $x \in \Omega \setminus J$ it holds that

3.9 $$\begin{aligned} {{\,\mathrm{supp}\,}}\partial _s\sigma _x = \{s,s+\pi \} \qquad \text{ for } \text{ some } s \in {\mathbb {R}}/2\pi {\mathbb {Z}}. \end{aligned}$$

### Proof

Let $\omega$ be a minimal Lagrangian representation and let $s,s' \in {\mathbb {R}}/2\pi {\mathbb {Z}}$; from the explicit expression of $\sigma _\omega$ we have that for ${\mathcal {L}}^1 \times \nu _{\min }$-a.e. $(t,x)\in (0,T)\times \Omega$ such that ${{\,\mathrm{supp}\,}}( \partial _s( \sigma _\omega )_{t,x}))\cap (s,s')\ne 0$ there exists $(\gamma ,t^-_\gamma ,t^+_\gamma )\in \Gamma _g$ such that

$$\begin{aligned} t \in (t^-_\gamma ,t^+_\gamma ), \qquad \gamma _x(t)=x, \qquad \text{ and } \qquad \big [\, \gamma _s(t-)\in (s,s') \quad \text{ or } \quad \gamma _s(t+)\in (s,s') \big ]. \end{aligned}$$

Given $s_1,s_2 \in \pi {\mathbb {Q}}/2\pi {\mathbb {Z}}$ with $s_1\ne s_2$ and $s_1 \ne s_2 + \pi$, we set

$$\begin{aligned} \delta _{s_1,s_2}= \frac{1}{3}\min \left\{ |s_1-s_2|, |s_1 + \pi -s_2| \right\} \end{aligned}$$

so that the intervals $I_1:=(s_1-\delta _{s_1,s_2}, s_1 + \delta _{s_1,s_2})$ , $I_2:=(s_2-\delta _{s_1,s_2}, s_2 + \delta _{s_1,s_2})$, $I_3:=(s_1+\pi -\delta _{s_1,s_2}, s_1 +\pi + \delta _{s_1,s_2})$ and $I_4:=(s_2+\pi -\delta _{s_1,s_2}, s_2 +\pi + \delta _{s_1,s_2})$ are pairwise disjoint and the distance between any two of these intervals is at least $\delta _{s_1,s_2}$. We denote by

$$\begin{aligned} E(s_1,s_2):= \left\{ (t,x) \in (0,T)\times \Omega : {{\,\mathrm{supp}\,}}( \partial _s(\sigma _\omega )_{t,x})) \cap I_j \ne \emptyset \text{ for } j=1,2,3,4 \right\} . \end{aligned}$$

It was shown in [13] that the constraint forces $\sigma$ to be $\pi$-periodic in *s*, in particular for ${\mathcal {L}}^1 \times \nu _{\min }$-a.e. $(t,x)\in (0,T)\times \Omega$ the support of $\partial _s \sigma _\omega$ is $\pi$-periodic. Therefore if $(t,x)\in (0,T)\times \Omega$ is such that (3.9) does not hold, then there exist four distinct points ${\bar{s}}_1,{\bar{s}}_2,{\bar{s}}_1 + \pi , {\bar{s}}_2 + \pi \in {\mathbb {R}}/2\pi {\mathbb {Z}}$ belonging to ${{\,\mathrm{supp}\,}}( \partial _s( \sigma _\omega )_{t,x})$. In particular ${\mathcal {L}}^1 \times \nu _{\min }$-a.e. $(t,x)\in (0,T)\times \Omega$ for which (3.9) does not hold belongs to

$$\begin{aligned} \bigcup _{s_1,s_2 \in \pi {\mathbb {Q}}/2\pi {\mathbb {Z}}} E(s_1,s_2). \end{aligned}$$

By the discussion at the beginning of the proof, we have that for ${\mathcal {L}}^1 \times \nu _{\min }$-a.e. $(t,x)\in E(s_1,s_2)$ and every $j=1,2,3,4$ there exists $(\gamma _j,t^-_{\gamma _j},t^+_{\gamma _j})\in \Gamma _g$ such that

$$\begin{aligned} t \in (t^-_{\gamma _j},t^+_{\gamma _j}), \qquad (\gamma _j)_x(t)=x, \qquad \text{ and } \qquad \big [\, (\gamma _j)_s(t-)\in I_j \quad \text{ or } \quad (\gamma _j)_s(t+)\in I_j) \big ]. \end{aligned}$$

We show that if $(t,x) \in E(s_1,s_2)$, then *x* is not a vanishing mean oscillation point of *m*. Let us assume by contradiction that *x* is a VMO point of *m* and there exists $t \in (0,T)$ such that $(t,x)\in E(s_1,s_2)$; by applying Lemma 3.1 for every $j=1,2,3,4$ there exists ${\bar{s}}_j \in I_j$ such that

$$\begin{aligned} \liminf _{r\rightarrow 0} \frac{{\mathscr {L}}^2(\{x' \in B_r( x): e^{i {\bar{s}}_j}\cdot m(x') > -\delta _{s_1,s_2} \})}{r^2} \ge c\delta _{s_1,s_2}. \end{aligned}$$

Since it does not exist any value ${\bar{m}} \in {\mathbb {R}}^2$ with $|{\bar{m}}|=1$ such that ${\bar{m}} \cdot e^{i{\bar{s}}_j}> - \delta _{s_1,s_2}$ for every $j=1,2,3,4$, this proves that *x* is not a vanishing mean oscillation point of *m*. Thm 1.5 implies that ${\mathcal {H}}^1$-a.e. $x \in \Omega \setminus J$ is a VMO point of *m*, therefore since $\nu _{\min }\ll {\mathcal {H}}^1$, then the set of points $x \in \Omega \setminus J$ for which there exists $t\in (0,T)$ such that $(t,x)\in E(s_1,s_2)$ is $\nu _{\min }$-negligible.

Letting $s_1,s_2$ vary in $\pi {\mathbb {Q}}/2\pi {\mathbb {Z}}$, this proves the claim. $\square$

### Remark 3.3

Proposition 1.7 states for the measure $\sigma _0$ the same property we obtained here for a minimal kinetic measure $\sigma$. Although $\sigma _0$ is not always a minimal kinetic measure, the two statements are equivalent since $\nu _{\min }\le \nu _0 \ll \nu _{\min }$ and $\partial _s \sigma _0 = \partial _s \sigma$ (see the discussion in Lemma 2.4).

### Corollary 3.4

For every $m\in A(\Omega )$ there exists a unique minimal kinetic measure $\sigma _{\min }$ of *m*. In particular for every minimal Lagrangian representation $\omega$ of *m* on $\Omega ' \subset \Omega$ it holds that

$$\begin{aligned} \sigma _\omega = {\mathscr {L}}^1\llcorner (0,T)\otimes \sigma _{\min }\llcorner \Omega '. \end{aligned}$$

Moreover the disintegration of $\sigma _{\min }$ with respect to $\nu _{\min }$ has the following structure:

1. for $\nu _{\min }$-a.e. $x \in \Omega \setminus J$ it holds that
  $$\begin{aligned} (\sigma _{\min } )_x =\frac{1}{2} (\delta _{{\bar{s}}-\frac{\pi }{2}} + \delta _{{\bar{s}} + \frac{\pi }{2}}), \qquad \text{ or } \qquad (\sigma _{\min } )_x =-\frac{1}{2} (\delta _{{\bar{s}}-\frac{\pi }{2}} + \delta _{{\bar{s}} + \frac{\pi }{2}}) \end{aligned}$$
  for some $${\bar{s}} \in {\mathbb {R}}/2\pi {\mathbb {Z}}$$.
2. for $\nu _{\min }$-a.e. $x \in J$ let $m^+, m^-$ and ${\mathbf {n}}$ denote the traces and the normal to *J* at *x* as in Theorem 1.5 and let $\beta \in (0,\pi )$ and ${\bar{s}}\in {\mathbb {R}}/2\pi {\mathbb {Z}}$ be uniquely determined by
  $$\begin{aligned} m^+= e^{i({\bar{s}} +\beta )}, \qquad \text{ and } \qquad m^-= e^{i({\bar{s}} -\beta )}. \end{aligned}$$
  Then
  $$\begin{aligned} (\sigma _{\min } )_x = {\mathbf {n}}\cdot e^{i{\bar{s}}} {\bar{g}}_\beta (s-{\bar{s}}){\mathscr {L}}^1, \end{aligned}$$
  where ${\bar{g}}_\beta :{\mathbb {R}}/2\pi {\mathbb {Z}}\rightarrow {\mathbb {R}}$ is $\pi$-periodic and for every $s \in [0,\pi ]$ is defined by
  3.10 $$\begin{aligned} \!\!\!\!\!\!\!\!\!\!{\bar{g}}_\beta (s):= {\left\{ \begin{array}{ll} c(\beta ) \left[ (\sin s - \cos \beta )\mathbbm {1}_{[\pi /2-\beta ,\pi /2+\beta ]}(s) \right] &{} \text{ if } \beta \in (0,\pi /4] \\ c(\beta ) \left[ (\sin s - \cos \beta )\mathbbm {1}_{[\pi /2-\beta ,\pi /2+\beta ]}(s) + \cos \beta - \frac{\sqrt{2}}{2}\right] &{} \text{ if } \beta \in (\pi /4,\pi /2] \\ {\bar{g}}_{\pi -\beta }(s) &{} \text{ if } \beta \in (\pi /2,\pi ),\nonumber \end{array}\right. }\\ \end{aligned}$$
  and where $c(\beta ) >0$ is such that
  $$\begin{aligned} \int _0^{2\pi }\left| {\bar{g}}_\beta (s)\right| ds = 1. \end{aligned}$$

### Proof

In particular let $\sigma$ be a minimal kinetic measure; since $\sigma$ is $\pi$-periodic in the variable *s*, it follows from Proposition 3.2 that for $\nu _{\min }$-a.e. $x \in \Omega \setminus J$ it holds that

$$\begin{aligned}&\sigma _x =\frac{1}{2+2\pi c} (\delta _{{\bar{s}}-\frac{\pi }{2}} + \delta _{{\bar{s}} + \frac{\pi }{2}} + c {\mathscr {L}}^1), \qquad \text{ or }\\&\sigma _x =-\frac{1}{2+ 2\pi c} (\delta _{{\bar{s}}-\frac{\pi }{2}} + \delta _{{\bar{s}} + \frac{\pi }{2}} +c {\mathscr {L}}^1) \end{aligned}$$

for some ${\bar{s}} \in {\mathbb {R}}/2\pi {\mathbb {Z}}$ and some $c \in {\mathbb {R}}$ depending on *x*. The necessary and sufficient condition (2.7) for minimality trivially implies $c=0$. By Theorem 1.5 and (1.9) it holds that

$$\begin{aligned} {\mathbf {n}}\cdot \left( \Phi (m^+)-\Phi (m^-) \right) {\mathscr {H}}^1\llcorner J = (p_x)_\sharp \left( - \partial _s\psi _\Phi \sigma \llcorner J\times {\mathbb {R}}/2\pi {\mathbb {Z}}\right) . \end{aligned}$$

The following identity was obtained in Sect. 4.2 of [13]: for every $\beta \in [0,\pi /2]$ it holds that

3.11 $$\begin{aligned} e_1 \cdot (\Phi (e^{i\beta })-\Phi (e^{-i\beta })) = - \int _0^{2\pi }g_\beta (s) \partial _s\psi _\Phi (s)ds, \end{aligned}$$

where $g_\beta :{\mathbb {R}}/2\pi {\mathbb {Z}}\rightarrow {\mathbb {R}}$ is a $\pi$-periodic defined by

$$\begin{aligned} g_\beta (s) = (\sin s - \cos \beta )\mathbbm {1}_{[\pi /2-\beta ,\pi /2+\beta ]}(s) - \frac{2}{\pi }(\sin \beta - \beta \cos \beta ) \qquad \forall s \in [0,\pi ]. \end{aligned}$$

Observe that the constraint $\mathrm {div}\, m = 0$ implies that for ${\mathscr {H}}^1$-a.e. $x\in J$ it holds $m^+\cdot {\mathbf {n}} = m^-\cdot {\mathbf {n}}$. Therefore, with the notation introduced in the statement, we have ${\mathbf {n}} = \pm e^{i{\bar{s}}}$. We prove (3.10) first in the case $\beta \in [0,\pi /2]$.

Choosing ${\tilde{\Phi }}$ such that $\psi _{{\tilde{\Phi }}}(s) = \psi _\Phi (s + {\bar{s}})$, we deduce from (3.11) that

$$\begin{aligned} {\mathbf {n}}\cdot \left( \Phi (m^+)-\Phi (m^-) \right) =&~ \left( {\mathbf {n}}\cdot e^{i{\bar{s}}}\right) e^{i{\bar{s}}}\cdot \left( \Phi \left( e^{i({\bar{s}} + \beta ) }\right) -\Phi \left( e^{i({\bar{s}} - \beta ) }\right) \right) \\ =&~ \left( {\mathbf {n}}\cdot e^{i{\bar{s}}}\right) e^{i{\bar{s}}}\cdot \int _{-\beta }^\beta \psi _{\Phi }\left( s+{\bar{s}}+ \frac{\pi }{2}\right) e^{i \left( s + {\bar{s}} +\frac{\pi }{2}\right) }ds \\ =&~ \left( {\mathbf {n}}\cdot e^{i{\bar{s}}}\right) e_1 \cdot \int _{-\beta }^{\beta } \psi _{\Phi } \left( s+{\bar{s}}+\frac{\pi }{2}\right) e^{i \left( s +\frac{\pi }{2}\right) }ds \\ =&~ \left( {\mathbf {n}}\cdot e^{i{\bar{s}}}\right) e_1 \cdot \left( {\tilde{\Phi }}\left( e^{i\beta }\right) -{\tilde{\Phi }}\left( e^{-i\beta }\right) \right) \\ =&~ - \left( {\mathbf {n}}\cdot e^{i{\bar{s}}}\right) \int _0^{2\pi }g_\beta (s)\psi '_{{\tilde{\Phi }}}(s)ds \\ =&~ - \left( {\mathbf {n}}\cdot e^{i{\bar{s}}}\right) \int _0^{2\pi }g_\beta (s-{\bar{s}})\psi '_{\Phi }(s)ds. \end{aligned}$$

This shows that for $\nu _{\min }$-a.e. $x \in J$ with $\beta \in (0,\pi /2)$ there exist two constants $c_1>0$ and $c_2 \in {\mathbb {R}}$ such that $\sigma _x= c_1(g_\beta (\cdot -{\bar{s}}) + c_2) {\mathscr {L}}^1$. It is a straightforward computation to check that the choice in (3.10) is the unique that satisfies the constraint in (2.7). In particular $\sigma _x$ is uniquely determined for $\nu _{\min }$-a.e. $x \in J$ such that $\beta \in (0,\pi /2)$.

The case $\beta \in (\pi /2,\pi )$, can be reduced to the previous case exchanging $m^+$ with $m^-$, and therefore changing the sign of ${\mathbf {n}}$ and replacing ${\bar{s}}$ with ${\bar{s}} + \pi$. Since $\partial _s \psi _\Phi$ and $g_\beta$ for $\beta \in (0,\pi /2]$ are $\pi$-periodic, then the same computations as above leads to

$$\begin{aligned} {\mathbf {n}}\cdot \left( \Phi (m^+)-\Phi (m^-) \right) = - \left( {\mathbf {n}}\cdot e^{i{\bar{s}}}\right) \int _0^{2\pi }g_{\pi -\beta }(s-{\bar{s}})\partial _s\psi _{\Phi }(s)ds. \end{aligned}$$

Similarly the choice in (3.10) is the unique that satisfies the constraint (2.7). $\sigma _x$ being uniquely determined for $\nu _{\min }$-a.e. $x \in \Omega$, the measure $\sigma _{\min }$ is unique. $\square$

The following lemma links the jump set of the characteristic curves with the jump set of $m \in A(\Omega )$:

### Lemma 3.5

Let $m \in A(\Omega )$ and $\Omega '$ be a $W^{2,\infty }$ open set compactly contained in $\Omega$. Let moreover $\omega$ be a minimal Lagrangian representation of *m* on $\Omega '$. Then for $\omega$-a.e. $(\gamma ,t^-_\gamma ,t^+_\gamma ) \in \Gamma$ the following property holds: for every $t \in (t^-_\gamma , t^+_\gamma )$ such that $\gamma _s(t+)\ne \gamma _s(t-)$ it holds that $\gamma _x(t)\in J$.

### Proof

Since $\omega$ is a minimal Lagrangian representation, by Proposition 2.5 and Corollary 3.4 it holds that

$$\begin{aligned} \int _\Gamma |\sigma _\gamma | d\omega = |\sigma _\omega | = {\mathcal {L}}^1 \times |\sigma _{\min }| = {\mathcal {L}}^1 \times \left( \nu _{\min }\otimes |(\sigma _{\min })_x|\right) \end{aligned}$$

as measures in $(0,T)\times \Omega '\times {\mathbb {R}}/2\pi {\mathbb {Z}}$. By Corollary 3.4 it follows that for ${\mathcal {L}}^1\times \nu _{\min }$-a.e. $(t,x) \in (0,T)\times (\Omega \setminus J)$, it holds that

3.12 $$\begin{aligned} {{\,\mathrm{supp}\,}}\left( {\mathcal {L}}^1 \times |\sigma _{\min }|\right) _{t,x} \subset \{ {\bar{s}}, {\bar{s}} + \pi \} \end{aligned}$$

for some ${\bar{s}} \in {\mathbb {R}}/2\pi {\mathbb {Z}}$. Suppose by contradiction that there exists $G \subset \Gamma$ with $\omega (G)>0$ and a measurable function ${\tilde{t}} : G \rightarrow (0,T)$ such that for every $(\gamma ,t^-_\gamma ,t^+_\gamma )$ in *G* it holds that

$$\begin{aligned} {\tilde{t}} (\gamma ) \in (t^-_\gamma ,t^+_\gamma ), \qquad \gamma _s\left( {\tilde{t}}(\gamma )+\right) \ne \gamma _s\left( {\tilde{t}}(\gamma )+\right) , \qquad \text{ and } \qquad \gamma _x\left( {\tilde{t}}(\gamma )\right) \in \Omega '\setminus J. \end{aligned}$$

For every $(\gamma ,t^-_\gamma ,t^+_\gamma ) \in G$ we set

$$\begin{aligned}&{\tilde{\sigma }}_\gamma = {\mathcal {H}}^1\llcorner E^+_\gamma \left( {\tilde{t}}(\gamma )\right) - {\mathcal {H}}^1\llcorner E^-_\gamma \left( {\tilde{t}}(\gamma )\right) , \qquad \text{ where } \\&\quad E^\pm _\gamma \left( {\tilde{t}}(\gamma )\right) := \{ (t,x,s) \in E^\pm _\gamma : t = {\tilde{t}}(\gamma )\}, \end{aligned}$$

and $E^{\pm }_\gamma$ are defined in (2.4). Let ${\tilde{\sigma }}_\omega := \int _\Gamma |{\tilde{\sigma }}_\gamma |d\omega \in {\mathcal {M}}^+((0,T)\times \Omega '\times {\mathbb {R}}/2\pi {\mathbb {Z}})$; by definition we have ${\tilde{\sigma }}_\omega \le |\sigma _\omega |$. Let us denote by ${\tilde{\nu }}:=(p_{t,x})_\sharp {\tilde{\sigma }}_\gamma$. Then by definition of ${\tilde{\sigma }}_\omega$ we have that ${\tilde{\nu }}$ is concentrated on $(0,T)\times \Omega '\setminus J$ and for ${\tilde{\nu }}$-a.e. $(t,x) \in (0,T)\times \Omega '\setminus J$ there exist no ${\bar{s}} \in {\mathbb {R}}/2\pi {\mathbb {Z}}$ such that ${{\,\mathrm{supp}\,}}({\tilde{\sigma }}_\omega )_{t,x} \subset \{{\bar{s}},{\bar{s}} + \pi \}$. Since ${\tilde{\nu }}(\Omega ')>0$, this is in contradiction with (3.12). $\square$

### Solutions with a Single Vanishing Entropy

The goal of this section is to prove the following result about solutions with vanishing entropy production:

#### Proposition 3.6

Let $\Omega \subset {\mathbb {R}}^2$ be an open set and $m\in A(\Omega )$ be such that $\mathrm {div}\Sigma _{\varepsilon _1,\varepsilon _2}(m)=0$. Then *J* is contained in the union of countably many horizontal and vertical segments. Moreover $\nu _{\min }$ is concentrated on *J*.

The result follows from Proposition 3.2 and the following general result about BV functions for which we refer to [3, Proposition 3.92]:

#### Lemma 3.7

Let $f \in {{\,\mathrm{BV}\,}}((0,T);{\mathbb {R}})$ be continuous from the right. Then for every $E\subset {\mathbb {R}}$ at most countable it holds

$$\begin{aligned} \big |{\tilde{D}}f\big |\left( f^{-1}(E)\right) =0. \end{aligned}$$

#### Proof of Proposition 3.6

We recall from [13] that

$$\begin{aligned} \mathrm {div}\Sigma _{\varepsilon _1,\varepsilon _2}(m)= - 2(p_x)_\sharp \left[ \sin (2s)\sigma \right] . \end{aligned}$$

For $\nu _{\min }$-a.e. $x \in J$ it holds $n=\pm e^{i{\bar{s}}}$, therefore in order to show that *J* is contained in a countable union of horizontal and vertical segments, it is sufficient to observe that for every $\beta \in (0,\pi )$ it holds that

3.13 $$\begin{aligned} \int _{{\mathbb {R}}/2\pi {\mathbb {Z}}}g_{\beta }(s-{\bar{s}})\sin (2s) ds = 0 \qquad \Longrightarrow \qquad {\bar{s}} \in \frac{\pi }{2}{\mathbb {Z}}. \end{aligned}$$

This can be proven directly by using the explicit expression of $g_\beta$ in (3.10). Alternatively, we refer to [6, Lemma 2.4], where the authors show that for $m\in A(\Omega )\cap BV(\Omega )$ it holds that

$$\begin{aligned} |\mathrm {div}\, \Sigma _{\varepsilon _1,\varepsilon _2}(m)| \llcorner J = \frac{1}{3}\cos (2\alpha ) |m^+ - m^-|^3 {\mathcal {H}}^1 \llcorner J, \end{aligned}$$

where $\alpha \in {\mathbb {R}}/2\pi {\mathbb {Z}}$ is such that $n = \pm e^{i\left( \alpha + \frac{\pi }{4} \right) }$. Theorem 1.5 implies that the same computation is valid for every $m \in A(\Omega )$. Since $\cos (2\alpha )= 0 \Rightarrow \alpha \in \frac{\pi }{4} + \frac{\pi }{2}{\mathbb {Z}}$, then $\mathrm {div}\Sigma _{\varepsilon _1,\varepsilon _2}(m)=0$ implies that $n = e^{i {\bar{s}}}$ with ${\bar{s}} \in \frac{\pi }{2}{\mathbb {Z}}$ a.e. with respect to the measure $\nu _{\min }\llcorner J$.

Now we prove that $\nu _{\min }$ is concentrated on *J*: by Corollary 3.4, for $\nu _{\min }$-a.e. $x \in \Omega \setminus J$ it holds that

$$\begin{aligned} \int _{{\mathbb {R}}/2\pi {\mathbb {Z}}}\sin (2s) d( \delta _{{\bar{s}}} + \delta _{{\bar{s}} + \pi })=0, \end{aligned}$$

which trivially implies ${\bar{s}} \in \frac{\pi }{2}{\mathbb {Z}}$. By Lemma 3.5, we have

$$\begin{aligned} {\mathscr {L}}^1\times \nu _{\min }\llcorner (\Omega \setminus J) \le \int _\Gamma (\gamma _x)_\sharp |{\tilde{D}}_t \gamma _s|\left( \gamma _s^{-1}\left( \frac{\pi }{2}{\mathbb {Z}}\right) \right) d\omega (\gamma ) = 0, \end{aligned}$$

where in the last equality we used Lemma 3.7. $\square$

#### Remark 3.8

The same argument shows that, in order to prove that $\nu _{\min }$ is concentrated on *J*, the assumption $\mathrm {div}\,\Sigma _{\varepsilon _1,\varepsilon _2}(m)=0$ can be replaced with $\mathrm {div}\,\Phi (m)=0$ for any $\Phi \in {\mathcal {E}}_\pi$ such that $\{s:\partial _s \psi _\Phi (s)=0\}$ is at most countable.

## Uniqueness of Minimizers on Ellipses

The goal of this section is to prove Theorem 1.8. Since the functional ${\tilde{F}}_0$ is invariant by rotations, then we will assume without loss of generality that the major axis of the ellipse is parallel to *x*-axis in the plane.

The next result is essentially contained in [16] (see also [15]); for completeness, we give the proof here.

### Proposition 4.1

Let ${\bar{u}}^\delta$ be defined as in Theorem 1.8. Then ${\bar{u}}^\delta$ is a minimizer of ${\tilde{F}}_0(\cdot , \Omega _\delta )$ in $\Lambda ^0_\delta (\Omega )$. Moreover, for every minimizer $u^\delta$ of ${\tilde{F}}_0(\cdot , \Omega _\delta )$ in $\Lambda ^0_\delta (\Omega )$ the function $m = \nabla ^\perp u^\delta$ satisfies

$$\begin{aligned} \mathrm {div}\Sigma _{\varepsilon _1,\varepsilon _2}(m)= 0 \qquad \text{ and } \qquad \mathrm {div}\Sigma _{e_1,e_2}(m)\ge 0 \qquad \text{ in } {\mathcal {D}}'(\Omega _\delta ). \end{aligned}$$

### Proof

In [2], the authors noticed that for every $u \in A(\Omega _\delta )$ it holds that

4.1 $$\begin{aligned} \begin{aligned} {\tilde{F}}_0(u,\Omega _\delta ) =&\left\| \left( \begin{array}{c} \text {div} \Sigma _{e_1,e_2}(\nabla ^\perp u) \\ \text {div} \Sigma _{\varepsilon _1,\varepsilon _2}(\nabla ^\perp u) \end{array} \right) \right\| (\Omega _\delta ) \\ \ge&~ \left( \left( \left| \mathrm {div}\Sigma _{e_1,e_2}(\nabla ^\perp u)\right| (\Omega _\delta )\right) ^2 + \left( \left| \mathrm {div}\Sigma _{\varepsilon _1,\varepsilon _2}(\nabla ^\perp u)\right| (\Omega _\delta )\right) ^2 \right) ^{\frac{1}{2}}. \end{aligned} \end{aligned}$$

Let us denote by ${\bar{m}} := \nabla ^\perp {\bar{u}}^\delta$. Since for every $u \in \Lambda _\delta (\Omega )$ it holds $\nabla ^\perp u = {\bar{m}}$ in $S_\delta$, then it follows from (4.1) that

4.2 $$\begin{aligned} \begin{aligned} {\tilde{F}}_0(u,\Omega _\delta ) \ge&~ \left( \left( \left| \mathrm {div}\Sigma _{\varepsilon _1,\varepsilon _2}(\nabla ^\perp u)\right| (\Omega _\delta )\right) ^2 + \left( \left| \mathrm {div}\Sigma _{e_1,e_2}(\nabla ^\perp u)\right| (\Omega _\delta )\right) ^2\right) ^{\frac{1}{2}} \\ \ge&~ \mathrm {div}\Sigma _{e_1,e_2}(\nabla ^\perp u)(\Omega _\delta ) \\ =&~ \int _{\partial \Omega _\delta } \Sigma _{e_1,e_2}(\nabla ^\perp u) \cdot n d{\mathcal {H}}^1 \\ =&~ \mathrm {div}\Sigma _{e_1,e_2}({\bar{m}})(\Omega _\delta ) \\ =&~ {\tilde{F}}_0({\bar{u}}^\delta ,\Omega _\delta ), \end{aligned} \end{aligned}$$

where in the last equality we used $\mathrm {div}\Sigma _{\varepsilon _1,\varepsilon _2}({\bar{m}})= 0$ and $\mathrm {div}\Sigma _{e_1,e_2}({\bar{m}})\ge 0$. This shows, in particular, that ${\bar{u}}^\delta$ is a minimizer of ${\tilde{F}}_0(\cdot ,\Omega _\delta )$ in $\Lambda ^0_\delta (\Omega )$. Moreover for every minimizer *u* of ${\tilde{F}}_0(\cdot ,\Omega _\delta )$ in $\Lambda ^0_\delta (\Omega )$, the inequality in (4.2) is an equality and this completes the proof. $\square$

### Theorem 4.2

Let $\Omega$ be an ellipse, and $m \in A_\delta (\Omega )$ be such that

4.3 $$\begin{aligned} \mathrm {div}\Sigma _{\varepsilon _1,\varepsilon _2}(m)= 0, \qquad \text{ and } \qquad \mathrm {div}\Sigma _{e_1,e_2}(m)\ge 0. \end{aligned}$$

Then

4.4 $$\begin{aligned} m\llcorner \Omega = \nabla ^\perp {{\,\mathrm{dist}\,}}(\cdot , \partial \Omega ). \end{aligned}$$

### Proof

The proof is divided into three steps: in Step 1 we link the assumptions in (4.3) with the sign of $\partial _s \sigma _{\min }$ relying on Corollary 3.4 and Proposition 3.6. Then we will prove in Step 2 that the entropy defect measures of every *m* as in the statement are concentrated on the axis of the ellipse. We finally prove in Step 3 that this last condition forces *m* to satisfy (4.4).

*Step 1*. Let $m \in A_\delta (\Omega )$ be as in the statement and $\sigma _{\min }$ be its minimal kinetic measure. Then, for every $\phi \in C^1_c(\Omega _\delta \times {\mathbb {R}}/2\pi {\mathbb {Z}})$ such that $\phi \ge 0$ and

$$\begin{aligned} {{\,\mathrm{supp}\,}}\phi \subset \Omega _\delta \times \left( \left( 0,\frac{\pi }{2}\right) \cup \left( \pi , \frac{3}{2}\pi \right) \right) , \end{aligned}$$

it holds that

$$\begin{aligned} \langle \partial _s\sigma _{\min }, \phi \rangle = - \int _{\Omega \times {\mathbb {R}}/2\pi {\mathbb {Z}}} \partial _s\phi d\sigma _{\min } \ge 0. \end{aligned}$$

*Proof of Step 1*. Since $\mathrm {div}\Sigma _{\varepsilon _1,\varepsilon _2}(m)= 0$, it follows from Proposition 3.6 that for $\nu _{\min }$-a.e. $x \in J$ the normal to *J* at *x* is ${\mathbf {n}}(x)=e^{is(x)}$ for some $s(x) \in \frac{\pi }{2}{\mathbb {Z}}$. Up to exchange $m^+$ and $m^-$, we can therefore assume without loss of generality that ${\mathbf {n}} (x) = (1,0)$ or ${\mathbf {n}}(x)=(0,1)$ for $\nu _{\min }$-a.e. $x \in J$. We denote by $J_h\subset J$ the points for which ${\mathbf {n}}=(0,1)$ and $J_v \subset J$ the points with ${\mathbf {n}}(x)=(1,0)$. We consider these two cases separately.

If $${\mathbf {n}}(x)=(0,1)$$, then

$$\begin{aligned} \left( \mathrm {div}\Sigma _{e_1,e_2}(m)\right) \llcorner J_h= \frac{1}{3} \left( (m^+_1)^3(x) - (m^-_1)^3(x) \right) {\mathscr {H}}^1\llcorner J_h, \end{aligned}$$

therefore $m^+_1(x)=-m^-_1(x)>0$ for $\nu$-a.e. $x \in J_h$. In particular, using the same notation as in Corollary 3.4, we have ${\bar{s}} = \frac{3}{2}\pi$. We observe that by the definition of ${\bar{g}}_\beta$ in (3.10), for every $\beta \in (0,\pi )$ it holds $\partial _s {\bar{g}}_\beta (s) \ge 0$ for ${\mathcal {L}}^1$- a.e. $s \in \left( 0,\frac{\pi }{2}\right) \cup \left( \pi , \frac{3}{2}\pi \right)$ and $\partial _s {\bar{g}}_\beta (s) \le 0$ for ${\mathcal {L}}^1$- a.e. $s \in \left( \frac{\pi }{2},\pi \right) \cup \left( \frac{3}{2}\pi ,2\pi \right)$. In particular for every $\beta \in (0,\pi )$ and ${\mathcal {L}}^1$- a.e. $s \in \left( 0,\frac{\pi }{2}\right) \cup \left( \pi , \frac{3}{2}\pi \right)$ it holds that

$$\begin{aligned} \left( {\mathbf {n}} \cdot {e^{i{\bar{s}}}}\right) \partial _s {\bar{g}}_{\beta }(s-{\bar{s}}) = {-} \partial _s {\bar{g}}_{\beta }(s-{\bar{s}}) \ge 0. \end{aligned}$$

Similarly, if ${\mathbf {n}}=(1,0)$, then

$$\begin{aligned} \left( \mathrm {div}\Sigma _{e_1,e_2}(m)\right) \llcorner J_v= \frac{1}{3} \left( (m^+_2)^3(x) - (m^-_2)^3(x) \right) {\mathscr {H}}^1\llcorner J_v, \end{aligned}$$

therefore $m^+_2(x)=-m^-_2(x)>0$ for $\nu _{\min }$-a.e. $x \in J_v$. In particular ${\bar{s}} = 0$ so that for every $\beta \in (0,\pi )$ and ${\mathcal {L}}^1$- a.e. $s \in \left( 0,\frac{\pi }{2}\right) \cup \left( \pi , \frac{3}{2}\pi \right)$ it holds that

$$\begin{aligned} \left( {\mathbf {n}} \cdot {e^{i{\bar{s}}}}\right) \partial _s {\bar{g}}_{\beta }(s-{\bar{s}}) =\partial _s {\bar{g}}_{\beta }(s) \ge 0. \end{aligned}$$

Therefore by Corollary 3.4, it follows that

$$\begin{aligned} \langle \partial _s\sigma _{\min }, \phi \rangle = \int _\Omega \int _0^{2\pi } \left( {\mathbf {n}} \cdot {e^{i{\bar{s}}}}\right) {\bar{g}}_\beta '(s-{\bar{s}}) \phi ds d\nu _{\min } \ge 0. \end{aligned}$$

*Step 2*. We prove that $\nu _{\min }$ is concentrated on the axis of the ellipse.

Let us denote by

$$\begin{aligned} \Omega = \left\{ x \in {\mathbb {R}}^2 : x_1^2 + a x_2^2 < r^2\right\} \end{aligned}$$

with $r>0$ and $a \ge 1$. Let us assume by contradiction that $\nu _{\min } ( J_h \cap \{x \in {\mathbb {R}}^2 : x_2>0\})>0$. Then there exists $b>0$ such that $\nu _{\min } (\{x \in J_h : x_2=b\})>0$. By the analysis in the proof of Step 1 there exists $A \subset {\mathbb {R}}$ such that ${\mathscr {L}}^1(A)>0$ and for ${\mathscr {H}}^1$-a.e. $x \in A \times \{b\}$ it holds $m^-_1(x)<0$. In particular we can choose $\alpha \in (\pi , 3\pi /2)$ such that

4.5 $$\begin{aligned}&|\tan \alpha | \le \frac{b}{2(r+\delta )} \qquad \text{ and } \nonumber \\&\eta := {\mathscr {H}}^1 \left( \left\{ x \in \Omega \cap J_h : x_2=b \text{ and } e^{i\alpha }\cdot m^-(x)>0\right\} \right) >0. \end{aligned}$$

Let ${\bar{x}}_1>0$ be such that $({\bar{x}}_1,b)\in \partial \Omega _\delta$ and denote by

**Fig. 1:**
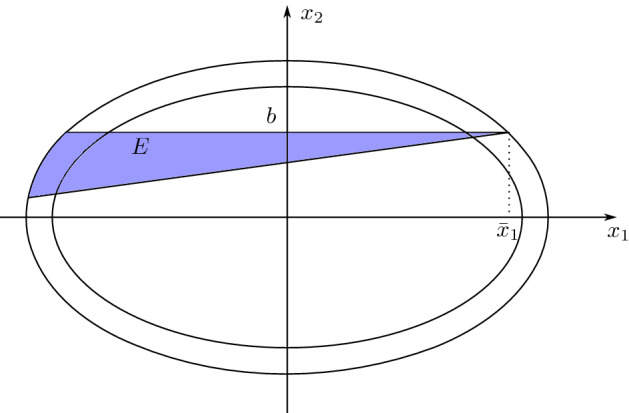
The figure illustrates the definition of *E* in (4.6)

4.6 $$\begin{aligned} E:=\{x \in \Omega _\delta : x_2 \in (g(x_1),b)\}, \end{aligned}$$

where $g(x_1)= \tan (\alpha ) (x_1-{\bar{x}}_1) + b$. The first constraint in (4.5) implies that $E\subset \{ x_2>0\}$ (see Fig. 1).

We consider the following Lipschitz approximation of the characteristic function of *E*:

$$\begin{aligned} \psi _\varepsilon ( x ) = {\left\{ \begin{array}{ll} 0 &{} \text{ if } x \notin E \\ \min \left\{ 1, \frac{1}{\varepsilon }{{\,\mathrm{dist}\,}}(x,\partial E) \right\} &{} \text{ if } x \in E. \end{array}\right. } \end{aligned}$$

We moreover consider $\rho \in C^\infty _c(\pi + \frac{\alpha -\pi }{2},\alpha )$ such that $\rho \ge 0$ and $\int _{\mathbb {R}}\rho (s) ds =1$ and we test (1.7) with $\varphi _\varepsilon (s,x) = \psi _\varepsilon (x) \rho (s)$. If $\varepsilon < \delta$, then the choice of $\alpha$ in (4.5) and of $\rho$ implies that

$$\begin{aligned} \{(x,s)\in & {} \Omega _\delta \times {{\,\mathrm{supp}\,}}(\rho ) :e^{is}\cdot \nabla _x \psi _\varepsilon< 0 \} \subset \{(x,s) \in (\Omega _\delta \setminus \Omega ) \times {{\,\mathrm{supp}\,}}(\rho ) :\\&x_2>0 \text{ and } x_1<0\}. \end{aligned}$$

Since $m= {\bar{m}}$ on $\Omega _\delta \setminus \Omega$, then for ${\mathscr {L}}^2 \times {\mathscr {L}}^1$-a.e. $(x,s) \in (\Omega _\delta \setminus \Omega ) \times {{\,\mathrm{supp}\,}}(\rho )$ it holds $\chi (x,s)=\mathbbm {1}_{e^{is}\cdot m(x)>0}=0$. In particular, by the second condition in (4.5), we have

$$\begin{aligned} \begin{aligned}&\liminf _{\varepsilon \rightarrow 0}\int _{\Omega \times {\mathbb {R}}/2\pi {\mathbb {Z}}} e^{is}\cdot \nabla _x \psi _\varepsilon (x) \rho (s) \chi (x,s) ds dx \\&\quad \ge ~ \int _{\{x \in \Omega :x_2=b\}\times {\mathbb {R}}/2\pi {\mathbb {Z}}} (-\sin s) \rho (s) \mathbbm {1}_{e^{is} \cdot m^-(x)>0} (x) ds d{\mathcal {H}}^1(x) \\&\quad \ge ~ \eta \sin \left( \frac{\alpha -\pi }{2}\right) \\&\quad >~ 0. \end{aligned} \end{aligned}$$

This contradicts Step 1, which implies that

$$\begin{aligned} \int _{\Omega \times {\mathbb {R}}/2\pi {\mathbb {Z}}} e^{is}\cdot \nabla _x \psi _\varepsilon (x) \rho (s) \chi (x,s)ds dx = - \langle \partial _s \sigma _{\min }, \rho \otimes \psi _\varepsilon \rangle \le 0. \end{aligned}$$

**Fig. 2:**
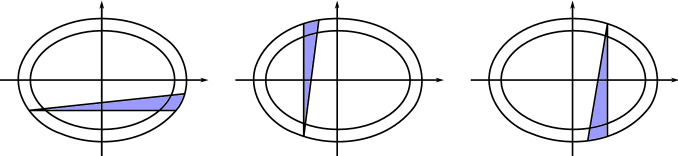
The regions in blue indicate the sets *E* to be considered in order to repeat the presented argument in the three cases not addressed in details

A similar argument excludes that $\nu _{\min }(\{x\in J_h: x_2=b\})>0$ if $b<0$ and that $\nu _{\min }(\{x\in J_v: x_1=a\})>0$ if $a\ne 0$; see Fig. 2 which illustrates the sets *E* that need to be considered in these cases.

*Step 3*. We prove that the unique $m\in A_\delta (\Omega )$ for which $\nu _{\min }$ is concentrated on the axis of the ellipse satisfies (4.4). In particular we show that $m = {\bar{m}}$ on

$$\begin{aligned} {\tilde{\Omega }}_\delta = \{ x \in \Omega _\delta : x_1<0, x_2>0\}, \end{aligned}$$

this being the argument for the other analogous quadrants.

Let ${\bar{x}} \in \Omega$ be a Lebesgue point of *m* and let ${\bar{s}}({\bar{x}}) \in (\pi /2,\pi )$ be such that

$$\begin{aligned} e^{i{\bar{s}}({\bar{x}})}= - \nabla {{\,\mathrm{dist}\,}}({\bar{x}}, \partial \Omega ). \end{aligned}$$

For every $s \in (\pi /2,\pi )$ let $t_s>0$ be the unique value such that

$$\begin{aligned} y_s:= {\bar{x}} + t_s e^{is} \in \partial \Omega _{\delta /2}\cap {\tilde{\Omega }}_\delta . \end{aligned}$$

**Fig. 3:**
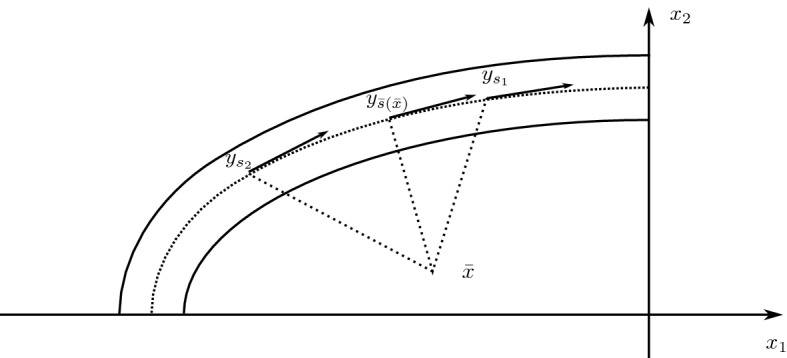
The picture represents the points $y_{s_1},y_{{\bar{s}}({\bar{x}})}, y_{s_2}$, while the arrows represent the values of ${\bar{m}}$ at these points

By elementary geometric considerations (see Fig. 3) the following properties hold:

1. $${\bar{m}} (y_s)\cdot e^{is} >0$$ for every $$s \in (\pi /2,{\bar{s}} ({\bar{x}}))$$;
2. $${\bar{m}} (y_s)\cdot e^{is} <0$$ for every $$s \in ({\bar{s}} ({\bar{x}}),\pi )$$.

In particular for every $\varepsilon \in \left( 0, \frac{1}{2}\min \{{\bar{s}}({\bar{x}}) -\pi /2, \pi - {\bar{s}}({\bar{x}})\}\right)$ there exists $r \in (0,\frac{\delta }{2})$ such that

1. for every $s \in ({\bar{s}}({\bar{x}}) - 2 \varepsilon , {\bar{s}} ({\bar{x}}) - \varepsilon )$ and every $y \in B_r(y_s)$ it holds ${\bar{m}} (y) \cdot e^{is}>0$;
2. for every $s \in ({\bar{s}}({\bar{x}}) + \varepsilon , {\bar{s}} ({\bar{x}}) +2 \varepsilon )$ and every $y \in B_r(y_s)$ it holds ${\bar{m}} (y) \cdot e^{is}<0$.

By Step 2 we have that

$$\begin{aligned} e^{is}\cdot \nabla _x \chi = 0 \qquad \text{ in } {\mathcal {D}}'({\tilde{\Omega }}_\delta ) \end{aligned}$$

therefore for ${\mathcal {L}}^1$-a.e. $s \in {\mathbb {R}}/2\pi {\mathbb {Z}}$ the sets $\left\{ x \in {\tilde{\Omega }}_\delta : e^{is}\cdot m(x)>0\right\}$ and $\left\{ x \in {\tilde{\Omega }}_\delta : e^{is}\cdot m(x)<0\right\}$ are invariant by translations in the direction $e^{is}$ up to negligible sets. Since $m = {\bar{m}}$ in ${\tilde{\Omega }}_\delta \setminus \Omega$, then it follows by the previous analysis that for every $\varepsilon >0$ there exists $r>0$ such that for ${\mathcal {L}}^2$-a.e. $x \in B_r({\bar{x}})$ the following two inequalities hold:

4.7 $$\begin{aligned} \begin{aligned} m(x)\cdot e^{is}>0&\qquad \text{ for } {\mathcal {L}}^1\text{-.a.e. } s \in ({\bar{s}}({\bar{x}}) - 2 \varepsilon , {\bar{s}} ({\bar{x}}) - \varepsilon ), \\ m(x)\cdot e^{is}<0&\qquad \text{ for } {\mathcal {L}}^1\text{-.a.e. } s \in ({\bar{s}}({\bar{x}}) +\varepsilon , {\bar{s}} ({\bar{x}}) +2 \varepsilon ). \end{aligned} \end{aligned}$$

The two conditions in (4.7) implies that for ${\mathcal {L}}^2$-a.e. $x \in B_r({\bar{x}})$ it holds $m(x)=e^{is(x)}$ for some $s(x) \in [{\bar{s}}({\bar{x}})- \pi /2 -\varepsilon , {\bar{s}}({\bar{x}})- \pi /2 +\varepsilon ]$. Since ${\bar{x}}$ is a Lebesgue point of *m*, letting $\varepsilon \rightarrow 0$, we obtain

$$\begin{aligned} m({\bar{x}})= {\bar{s}}({\bar{x}}) - \frac{\pi }{2} = {\bar{m}}({\bar{x}}). \end{aligned}$$

This concludes the proof. $\square$
